# Management of patients with an electrical storm or clustered ventricular arrhythmias: a clinical consensus statement of the European Heart Rhythm Association of the ESC—endorsed by the Asia-Pacific Heart Rhythm Society, Heart Rhythm Society, and Latin-American Heart Rhythm Society

**DOI:** 10.1093/europace/euae049

**Published:** 2024-04-08

**Authors:** Radosław Lenarczyk, Katja Zeppenfeld, Jacob Tfelt-Hansen, Frank R Heinzel, Thomas Deneke, Elena Ene, Christian Meyer, Arthur Wilde, Elena Arbelo, Ewa Jędrzejczyk-Patej, Avi Sabbag, Markus Stühlinger, Luigi di Biase, Marmar Vaseghi, Ohad Ziv, William-Fernando Bautista-Vargas, Saurabh Kumar, Narayanan Namboodiri, Benhur Davi Henz, Jose Montero-Cabezas, Nikolaos Dagres, Petr Peichl, Antonio Frontera, Stylianos Tzeis, Jose Luis Merino, Kyoko Soejima, Christian de Chillou, Roderick Tung, Lars Eckardt, Philippe Maury, Peter Hlivak, Larisa G Tereshchenko, Pipin Kojodjojo, Jacob Atié

**Affiliations:** Medical University of Silesia, Division of Medical Sciences, Department of Cardiology and Electrotherapy, Silesian Center for Heart Diseases, Skłodowskiej-Curie 9, 41-800 Zabrze, Poland; Department of Cardiology, Leiden University Medical Center, Leiden, The Netherlands; The Department of Cardiology, The Heart Centre, Copenhagen University Hospital, Rigshospitalet, Copenhagen, Denmark; The Department of Forensic Medicine, Copenhagen University Hospital, Rigshospitalet, Copenhagen, Denmark; Cardiology, Angiology, Intensive Care, Städtisches Klinikum Dresden Campus Friedrichstadt, Dresden, Germany; Clinic for Interventional Electrophysiology, Heart Center RHÖN-KLINIKUM Campus Bad Neustadt, Bad Neustadt an der Saale, Germany; Clinic for Electrophysiology, Klinikum Nuernberg, University Hospital of the Paracelsus Medical University, Nuernberg, Germany; Clinic for Interventional Electrophysiology, Heart Center RHÖN-KLINIKUM Campus Bad Neustadt, Bad Neustadt an der Saale, Germany; Division of Cardiology/Angiology/Intensive Care, EVK Düsseldorf, Teaching Hospital University of Düsseldorf, Düsseldorf, Germany; Department of Cardiology, Amsterdam UMC University of Amsterdam, Amsterdam, the Netherlands; Amsterdam Cardiovascular Sciences, Heart Failure and arrhythmias, Amsterdam, the Netherlands; Arrhythmia Section, Cardiology Department, Hospital Clínic, Universitat de Barcelona, Barcelona, Spain; IDIBAPS, Institut d'Investigació August Pi i Sunyer (IDIBAPS), Barcelona, Spain; Centro de Investigación Biomédica en Red de Enfermedades Cardiovasculares (CIBERCV), Madrid, Spain; Department of Cardiology, Congenital Heart Diseases and Electrotherapy, Silesian Centre for Heart Diseases, Zabrze, Poland; The Davidai Center for Rhythm Disturbances and Pacing, Chaim Sheba Medical Center, Tel Hashomer, Israel; School of Medicine, Faculty of Medical and Health Sciences, Tel Aviv University, Tel Aviv, Israel; Department of Internal Medicine III, Cardiology and Angiology, Medical University of Innsbruck, Innsbruck, Austria; Albert Einstein College of Medicine at Montefiore Hospital, New York, NY, USA; UCLA Cardiac Arrythmia Center, Division of Cardiology, Department of Medicine, University of California, Los Angeles, CA, USA; Case Western Reserve University, Cleveland, OH, USA; The MetroHealth System Campus, Cleveland, OH, USA; Cardiac Electrophysiology, Fundacion CardioInfantil La Cardio, Bogota, Colombia; Department of Cardiology, Westmead Hospital, Westmead Applied Research Centre, University of Sydney, Sydney, Australia; University of Science and Technology Kochi, Kerala, India; Instituto Brasilia de Arritmias-Hospital do Coração do Brasil-Rede Dor São Luiz, Brasilia, Brazil; Department of Cardiology, Leiden University Medical Center, Leiden, The Netherlands; German Heart Center of the Charite, Berlin, Germany; Department of Cardiology, IKEM, Prague, Czech Republic; Department of Electrophysiology, Great Metropolitan Hospital Niguarda, Milan, Italy; Cardoilogy Department, Mitera General Hospital, Hygeia Group, Athens, Greece; Arrhythmia and Robotic EP Unit, Hospital Universitario La Paz, Madrid, Spain; Department of Cardiovascular Medicine, Kyorin University Hospital, Tokyo, Japan; Department of Cardiology, University Hospital Nancy, Vandoeuvre les Nancy, France; Division of Cardiology, Cardiovascular Clinical Research, Banner Heart Institute, The University of Arizona College of Medicine-Phoenix, AZ, USA; Department of Cardiology – Electrophysiology, University of Münster, Münster, Germany; Cardiology, University Hospital Rangueil Toulouse, Toulouse, France; Department of Arrhythmias and Pacing, National Institute of Cardiovascular Diseases and the Slovak Medical University School of Medicine, Bratislava, Slovakia; Department of Cardiovascular Medicine, Heart, Vascular & Thoracic Institute, Department of Quantitative Health Sciences, Lerner Research Institute, Cleveland Clinic, Cardiology, USA; Asian Heart and Vascular Center, Singapore, Singapore; Hospital São Vicente – Rede Dor, Department of Cardiology, Federal University of Rio de Janeiro, Rio de Janeiro, Brazil

**Keywords:** Electrical storm, Arrhythmia, Ventricular tachycardia, Ventricular fibrillation, Sudden cardiac death, Consensus document

## Abstract

Electrical storm (ES) is a state of electrical instability, manifesting as recurrent ventricular arrhythmias (VAs) over a short period of time (three or more episodes of sustained VA within 24 h, separated by at least 5 min, requiring termination by an intervention). The clinical presentation can vary, but ES is usually a cardiac emergency. Electrical storm mainly affects patients with structural or primary electrical heart disease, often with an implantable cardioverter-defibrillator (ICD). Management of ES requires a multi-faceted approach and the involvement of multi-disciplinary teams, but despite advanced treatment and often invasive procedures, it is associated with high morbidity and mortality. With an ageing population, longer survival of heart failure patients, and an increasing number of patients with ICD, the incidence of ES is expected to increase. This European Heart Rhythm Association clinical consensus statement focuses on pathophysiology, clinical presentation, diagnostic evaluation, and acute and long-term management of patients presenting with ES or clustered VA.

## Graphical Abstract

**Graphical abstract:**
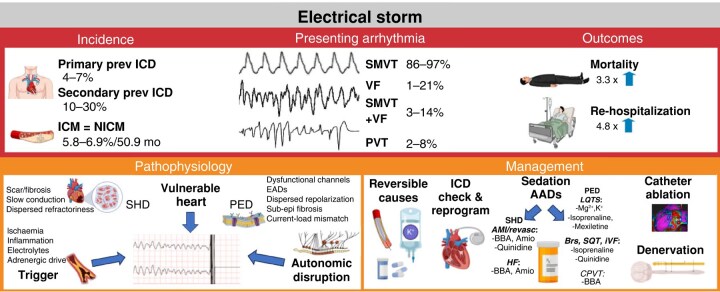

## Table of contents

1. Background
2. Aim of the document
3. Review of evidence
4. Definitions
5. Scope of the problem
6. Pathophysiological aspects
 6.1. The vulnerable heart—underlying structural heart disease
 6.2. The vulnerable heart—underlying primary electrical disease
 6.3. External precipitation factors or triggers
 6.4. The autonomic nervous system
7. Clinical presentation
8. Acute management
 8.1. Initial evaluation, assessment of external precipitating factors, and reversible causes
  8.1.1. History
  8.1.2. Twelve-lead electrocardiogram
  8.1.3. Documentation of ventricular arrhythmia and immediate implantable cardioverter-defibrillator interrogation
  8.1.4. Blood tests
  8.1.5. Coronary angiogram, cardiac imaging, and additional diagnostic studies
 8.2. Treatment
  8.2.1. Electrical storm with haemodynamic instability
  8.2.2. Disease-specific aspects
  8.2.3. Implantable cardioverter-defibrillator reprogramming
  8.2.4. Deep sedation and mechanical ventilation
   8.2.4.1. Agents used for (deep) sedation
  8.2.5. Pharmacotherapy for the acute management of electrical storm
   8.2.5.1. Structural heart disease
   8.2.5.2. Primary electrical disease
  8.2.6. Mechanical circulatory support
   8.2.6.1. Rescue therapy in acute haemodynamic decompensation because of refractory, haemodynamically intolerant ventricular arrhythmia
   8.2.6.2. Rescue therapy in peri-procedural acute haemodynamic decompensation because of refractory unstable ventricular arrhythmia in patients undergoing ventricular tachycardia ablation
   8.2.6.3. Prophylactical mechanical circulatory support implantation prior to ventricular tachycardia ablation in patients at high risk of developing haemodynamic instability
  8.2.7. Acute catheter ablation
  8.2.8. Autonomic modulation
   8.2.8.1. Stellate ganglion block
   8.2.8.2. Thoracic epidural anaesthesia
   8.2.8.3. Surgical/thoracoscopic sympathetic cardiac denervation
   8.2.8.4. Renal denervation
  8.2.9. Overdrive pacing
  8.2.10. Bailout strategies
9. Stabilized patient
 9.1. Long-term treatment
 9.2. Anti-arrhythmic drugs to prevent electrical storm recurrence
  9.2.1. Chronic anti-arrhythmic drug therapy in patients with structural heart disease
  9.2.2. Chronic pharmacotherapy in primary electrical disease
 9.3. Catheter ablation to prevent electrical storm recurrence
  9.3.1. Ischaemic heart disease
  9.3.2. Non-ischaemic cardiomyopathy
  9.3.3. Primary electrical disease
 9.4. Autonomic modulation
10. Psychological counselling
11. Special patient groups
 11.1. Patients with left ventricular assist device
 11.2. Patients with advanced heart failure
12. When not to perform interventional treatment
References

## 1. Background

Ventricular electrical storm (ES) is defined as three or more episodes of sustained ventricular arrhythmias (VAs) occurring within 24 h, separated by at least 5 min, requiring termination by an intervention.^1^ According to this definition, the clinical presentation can vary from asymptomatic or mildly symptomatic episodes of well-tolerated ventricular tachycardia (VT) to a life-threatening electrical instability, often aggravated by enhanced sympathetic tone. Similarly, clustered VAs, defined as ≥2 episodes of sustained VA within 3 months, can encompass a wide spectrum of clinical presentations.^2^ Electrical storm and clustered VA are frequently encountered in patients with ICDs. Most patients have underlying structural heart disease (SHD), with monomorphic sustained VT (MSVT) as the most common initiating arrhythmia. Electrical storm due to polymorphic VT (PVT) or primary ventricular fibrillation (VF) also occurs and is more likely in the setting of acute cardiac ischaemia or in patients with channelopathies [primary electrical diseases (PEDs)].^3^ Electrical storm with recurrent ICD shocks has been associated with psychological disorders, heart failure (HF), and increased mortality and is considered a cardiac emergency. Management of ES requires a multi-faceted approach and involvement of multi-disciplinary teams.

## 2. Aim of the document

In 2022, the European Society of Cardiology (ESC) released guidelines for management of patients with VA and the prevention of sudden cardiac death (SCD).^1^ In 2017, a document on the same topic was released by the Heart Rhythm Society (HRS), the American College of Cardiology, and the American Heart Association.^4,5^ A consensus statement addressing, specifically, catheter ablation (CA) of VA was issued by the HRS and the European Heart Rhythm Association (EHRA) in 2019, in collaboration with the Asia Pacific HRS (APHRS) and the Latin American HRS (LAHRS).^6^ This document aims to complement the prior ESC guidelines and consensus statements by providing detailed practical advice on diagnostic evaluation and acute and long-term management of patients presenting with ES or clustered VA. The definition of the different categories of advice is provided in *Table 1*. The used categories for strength of advice and supportive evidence are listed in *Table 2*. It should be emphasized that this EHRA Clinical Consensus statement is not intended as a guideline.

**Table 1:** Category of advice

Definition	Categories of advice
Evidence or general agreement that a given measure is clinically useful and appropriate	Advice TO DO
Evidence or general agreement that a given measure may be clinically useful and appropriate	May be appropriate TO DO
Evidence or general agreement that a given measure is not appropriate or harmful	Advice NOT TO DO
No strong advice can be given, lack of data, inconsistency of data	Area of uncertainty

**Table 2:** Strength of advice and strength of evidence

Strength of advice	Symbol
Clinical advice, based on robust published evidence	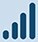
Clinical advice, based on consensus of the writing group	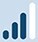
May be appropriate, based on published evidence	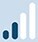
May be appropriate, based on consensus of the writing group	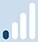
Areas of uncertainty	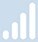
**Strength of evidence**	**Abbreviation**
Meta-analysis of RCTs	META
RCT	RCT
Observational studies	OBS
Expert opinion	OPN

META: evidence from >1 high-quality RCT or >1 meta-analyses of high-quality RCTs. RCT: evidence from 1 high-quality RCT, >1 moderate-quality RCTs, or meta-analyses of moderate-quality RCTs. OBS: observational or registries studies or meta-analyses of such studies. OPN: randomized, non-randomized, observational studies, or registries with limitations of design or execution. Meta-analyses of such studies. Physiological or mechanistic studies in human subjects. Consensus of expert opinion based on clinical experience. Case series.

RCT, randomized clinical trial.

## 3. Review of evidence

The EHRA Task Force prepared this consensus document with HRS, LAHRS, and APHRS representatives. Members of the Task Force were asked to review English-language literature across multiple databases, including PubMed, Embase, and the Cochrane Library, incorporating studies up to November 2023. Search was focused on all aspects of ES, including prevalence, pathophysiology, clinical manifestation, and management. Advices were constructed considering the strength of evidence supporting particular management or procedure and estimated patient outcomes. Potential sources of bias or factors that might have modified the results (e.g. number and characteristics of patients, duration of follow-up, patient groups’ heterogeneity, physician/patient preferences, etc) were identified and considered when establishing the strength of advices. In controversial or poorly evidenced areas, a consensus was reached by agreement of the expert group. Writing and reviewing were both iterative and collaborative, aligning with the principles of the Delphi process, and ensuring a broad range of expert opinions were considered. All advices were voted on using an online voting system, and consent of ≥80% of voters was required to accept the advice. The document underwent a thorough, two-round external review.

## 4. Definitions

## 5. Scope of the problem

The reported incidence of ES depends on the definition, studied population, and observation period.^7–10^ Electrical storm occurs in 10–30% of patients who have received an ICD for secondary prevention of SCD. The average time after device implantation to ES is 4–9 months.^7,11,12^ The incidence of ES is lower in patients who undergo ICD implantation for primary prevention of SCD and is estimated to be 4–7%, 18–24 months after ICD implantation.^8–10,12^ The reported incidence of clustered VA was 26% during 40 months in ICD recipients for primary or secondary SCD prevention.^2^

The incidence of ES is not significantly different in primary prevention ICD recipients with chronic coronary artery disease (cCAD) or non-ischaemic cardiomyopathy (NICM) during long-term follow-up (5.8 vs. 6.9%, respectively, within 50.9 ± 33.9 months after implantation).^10,13,14^ Cardiac resynchronization therapy (CRT) may influence the occurrence of ES. In a retrospective observational study of a mixed cohort of patients with cCAD and NICM, patients with CRT-defibrillator had a lower ES incidence compared with propensity-matched ICD recipients. However, in some patients with CRT, left ventricular (LV) pacing may cause ES and should be disabled in such cases. Nevertheless, CRT-responders had lower ES rates,^12^ which may be explained by the association between higher LV ejection fraction (LVEF) and lower risk of ES.^15^ With the growing number of patients with ICDs, the number of patients requiring ES management is likely to increase.

Data on ES in patients with PED are scarce and based on case reports or small series. Though ES incidence among PED patients seems to be low, increasing ICD use and improved survival are likely to also increase the rate of ES in this group.^16^ In patients with catecholaminergic PVT (CPVT), ICD shocks are sometimes ineffective and may trigger ES.^17^ Clustered VA and ES, according to the definition above, are more common in patients with known SHD and ICDs, but occasionally, ES may be the first manifestation of a previously not known cardiac disease.

Electrical storm is associated with high morbidity and mortality, particularly in the presence of additional factors (including amiodarone use, chronic kidney disease, and LVEF < 35%).^18–22^ Electrical storm was associated with a 2.5-fold higher risk of all-cause mortality compared with patients with sporadic episodes of VT and a 3.3-fold higher risk compared with patients without sustained VAs during follow-up.^13^ In a mixed cohort (cCAD and NICM) of ICD recipients, those who were admitted with an ES had a 4.8-fold higher risk of re-hospitalization and a 1.8-fold higher risk of major adverse cardiac events [defined as a composite of acute myocardial infarction (AMI), target vessel revascularization, and all-cause mortality], compared with patients with VT or VF but without ES.^20^ Clustered VA was also associated with a 2.7-fold increased risk of death compared with ICD recipients without arrhythmia, and mortality risk increased with higher VA burden (number of VA episodes in a cluster) and shorter cluster length (the time frame during which a specified number of VA events occurred).^2^ A 5-year survival free of death or heart transplant was 67 and 87%, respectively, in patients with and without clusters.^23^

## 6. Pathophysiological aspects

The pathophysiological mechanisms leading to ES are difficult to study in humans and differ depending on the underlying structural or electrical heart disease.

In a rabbit model, a complex interplay among enhanced sympathetic tone, calcium-related signalling abnormalities, dysregulation of protein phosphorylation, and a susceptible arrhythmogenic substrate was shown to contribute to VF episodes.^24^ It has been suggested that three key factors are prerequisite or contribute to ES, namely (i) a ‘vulnerable’ heart—which is a ‘conditio sine qua non’ of ES (i.e. the presence of a pre-existing heart disease that creates the necessary anatomical or electrical ‘substrate’), (ii) external precipitating factors or triggers, and (iii) a disruption of autonomic nervous system activity, particularly sympathetic activation. Although several external factors may be involved (see Sections 6.3. and 8.2.2.), a likely initiating trigger has, however, only been identified in 13% of ICD recipients with SHD.^7,25^ The role of the autonomic system in promoting and maintaining ES has been recognized.^26,27^ Genetic predisposition may play a role as well. Rare genetic variants in genes associated with inherited syndromes of sudden cardiac death may also predispose to ES in patients with cCAD.^28^ Early repolarization patterns have been described in patients with cCAD or NICM presenting with ES.^29^ A significant correlation between early repolarization patterns and VA recurrences in patients with idiopathic VF (IVF) could be observed.^30^

### 6.1. The vulnerable heart—underlying structural heart disease

Most patients presenting with ES or clustered VA have SHD. The critical VA substrate is typically related to areas of heterogeneous fibrosis, which can be observed in patients with cCAD and different cardiomyopathy phenotypes, including arrhythmogenic right ventricular cardiomyopathy (ARVC), dilated cardiomyopathy (DCM), and hypertrophic cardiomyopathy (HCM). Ventricular arrhythmia due to a diseased conduction system (e.g. Purkinje system-related) also needs to be recognized.^31^

Patients with SHD presenting with an ES are older, more often male, with a lower LVEF, advanced HF, and higher cardiovascular comorbidities compared with those without ES.^32,33^ In an observational study of ICD patients, chronic renal failure, depressed LVEF, and MSVT as index arrhythmia were independent predictors of an ES.^33^ Among patients who underwent radiofrequency CA (RFCA) for MSVT, those with history of ES had failed more anti-arrhythmic drugs (AADs), had more inducible VTs, and required longer procedure times and radiofrequency delivery time to control VT, suggesting a more complex or advanced VA substrate.^14^ In a study of patients with cCAD, ES patients had more diseased LV segments and more often anterior, septal, and apical endocardial LV scar. In contrast, a recent observational study of 152 consecutive patients with cCAD (43% with ES) undergoing VT ablation found no differences in the incidence of ES between patients after anterior and inferior AMI.^34^ In patients with NICM, the overall extent of the electroanatomical scar was similar, but lateral LV endocardial scar was more frequently observed in ES patients than in non-ES patients.^32^

### 6.2. The vulnerable heart—underlying primary electrical disease

All inherited arrhythmia syndromes carry a significant risk of SCD secondary to malignant VAs. In patients with long QT syndromes (LQTS) (acquired or congenital), torsade de pointes (TdP), with or without deterioration into VF, is the signature arrhythmia, and in the other arrhythmia syndromes, rapid PVT or VF is pertinent.^35^

In LQTS, adrenergic stimulation exerts genotype-specific effects.^36^ In LQT1, in which most arrhythmic events are triggered by exercise, sustained β stimulation enhances Ca^2+^ L-type inward current (I_CaL_), which is not counterbalanced by a slowly rectifying I_Ks_ potassium current, due to a dysfunctional pore-forming α-subunit. This results in the prolongation of action potential duration (APD). Sudden adrenergic surges (like during arousal) do not induce this effect due to the protective role of intact fast rectifying I_Kr_ current.^37,38^ On the contrary, in LQT2, in which VA is evoked commonly by sudden arousal (e.g. loud noise, startle), abrupt adrenergic drive causes imbalance between APD-prolonging I_CaL_ and dysfunctional I_Kr_ currents, with resulting prolongation in APD, whereas more sustained adrenergic stimulation (like during exercise) does not have such an effect due to the protective role of the intact countervailing I_Ks_ current.^39^ In CPVT, adrenergic stimulation plays an essential role in increasing calcium leak through ryanodine receptor Type 2, triggering delayed afterdepolarizations, and VAs,^40^ especially from Purkinje cells.^41^

In contrast, in Brugada syndrome (BrS) and early repolarization syndrome (ERS), bradycardia, beta-adrenergic blockade, or vagal stimulation and fever are considered pro-arrhythmic.^42–46^ Conventionally, an epicardial to endocardial repolarization gradient was assumed as one of the underlying mechanisms for arrhythmia initiation.^36,47,48^ More recently, microstructural abnormalities of the extracellular matrix (fibrosis), particularly within right ventricular subepicardial myocardium, manifesting as epicardial regions of abnormal, fractionated potentials, were found in patients with BrS and less frequently in ERS and IVF.^49–51^ This suggests the presence of subtle subepicardial cardiomyopathy, rather than purely electric disease.^52–56^. Subepicardial conduction in right ventricular outflow tract (RVOT) epicardial regions can be further compromised by impaired Na^+^ current due to the SCN5A pathogenic variant, Na^+^ channel blockade, or pacing, leading to slow/asynchronous conduction, areas of localized block, and creating large potential difference relative to the body of the right ventricle (RV; depolarization theory).^57^ Excitation failure due to current-to-load mismatch and localized RV epicardial activation delay may manifest as J-point/ST-segment elevation and predispose to re-entry and PVT/VF.^58^ Short QT (SQT) syndrome is a rare arrhythmogenic syndrome associated with several genetic mutations, including gain-of-function mutations of potassium channels and loss-of-function mutations of calcium and sodium channels, resulting in a shortened repolarization phase of the action potential and transmural repolarization dispersion.^59,60^

The diagnosis of idiopathic VF requires exclusion of an underlying structural, channelopathic, metabolic, or toxicological aetiology.^1^ Detailed invasive mapping studies have revealed subtle microstructural changes, leading to depolarization abnormalities that allow for re-entry and VF in a subset of patients diagnosed with idiopathic VF.^51,54^ In patients with idiopathic VF, ES can be triggered by premature ventricular complexes (PVCs), originating from the Purkinje system, RVOT, or the papillary muscle.^61^ Of importance, overlapping mechanism may exist that contribute to arrhythmogenicity. For example, in Purkinje-related idiopathic VF, repolarization abnormalities may play an important role, such as in overexpression of dipeptidyl peptidase-like protein-6.^62,63^

### 6.3. External precipitation factors or triggers

External and reversible factors that may provoke an ES (see Section 8.2.2.) include acute myocardial ischaemia, electrolyte imbalance (e.g. hypokalaemia), fever (e.g. in BrS), hypothermia (e.g. in ERS), hormonal factors (e.g. hyperthyirodism), sepsis, starvation, decompensated HF, physical exertion or emotional stress, and non-compliance to medical advices/or medications. However, a specific trigger is only identified in the minority of cases.^25^

### 6.4. The autonomic nervous system

Structural heart disease is associated with an imbalance between the sympathetic and parasympathetic limbs of the autonomic nervous systems. Myocardial infarction (MI) and HF result in neural remodelling. In animal models and humans post-MI, sympathetic remodelling occurs due to injury and denervation of sympathetic fibres within scar areas, subsequent localized nerve sprouting in the border zone regions, and loss of efferent sympathetic nerves in non-infarcted areas, distal (apical) to the infarcted area.^64–66^ This remodelling is followed by increased systemic but decreased cardiac levels of catecholamines and regional super-sensitivity to catecholamines in the scarred (denervated) areas.^66^ Changes in the parasympathetic limb are less well defined, with diminished acetylcholine release at the nerve–myocyte interface caused by the high norepinephrine levels and other sympathetic cotransmitters (galanin and neuropeptide Y) as one of the suspected mechanisms.^67,68^ The sympathetic/parasympathetic imbalance promotes arrhythmogenesis in many ways: by both shortening and creating heterogeneities of APD, by increasing dispersion of repolarization (and consequently of refractoriness), and by promoting early and late afterdepolarizations.^69^

In PED patients, the role of the autonomic system is crucial and disease -specific (see Sections 6.2. and 9.2.2.).

## 7. Clinical presentation

**Figure 1:**
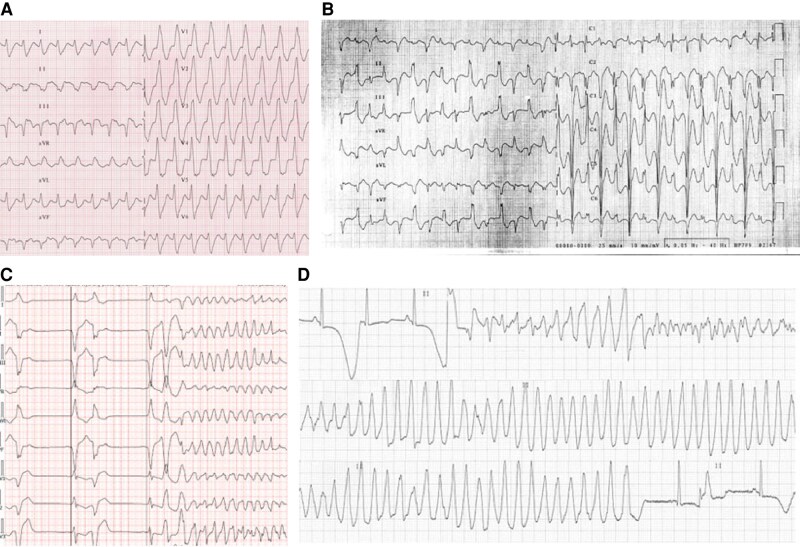
Types of ventricular arrhythmias: (*A*) monomorphic VT, (*B*) bidirectional VT, (*C*) polymorphic VT initiated by shortly coupled monomorphic premature ventricular complexes, (*D*) torsade de pointes in long QT syndrome; macroscopic T-wave alternans prior to VT. VT, ventricular tachycardia.

Patients with clustered VA or ES, according to the definition used, can be asymptomatic or can present with a broad spectrum of symptoms. Symptoms can range from palpitations due to tolerated VT [often with VT cycle length (CL) below ICD detection] or VT terminated by anti-tachycardia pacing (ATP), through pre-syncope or syncope despite VT termination by ATP or ICD shocks, to haemodynamically unstable VT or VF, requiring advanced cardiovascular life support (ACLS), multiple external cardioversions (CVs)/defibrillations (DFs), or even arrhythmic death.^70^ Arrhythmias responsible for an ES are MSVT in the majority of cases (86–97%), followed by primary VF (1–21%), both MSVT/VF (3–14%) and PVT (2–8%) (*Table 3* and *Figure 1*). In more than half of patients with an ES, the intervals between VT/VF episodes are shorter than 1 h.^7,9,11,25,33,71–73^

**Table 3:** Definitions for VA subtypes

Definitions of VA	
Incessant VT	Continuous sustained VT that recurs promptly despite repeated intervention for termination over several hours
Sustained VT	VT for at least 30 s or which requires an acute intervention for termination
Non-sustained VT	Run of consecutive ventricular beats persisting for at least 3 beats to <30 s duration
Monomorphic VT	VT with the same QRS morphology from beat to beat
PVT	VT showing an abrupt morphological change of the 12-lead ECG VT morphology during an ongoing VT episode
Bidirectional VT	VT with beat-to-beat alternation of the frontal QRS axis
PVT	VT with continually changing QRS morphology
TdP	Subtype of a PVT in the context of QT prolongation, with continually changing QRS complexes that appear to spiral around the baseline of the ECG lead in a sinusoidal pattern
Short coupled ventricular complexes	A PVC that interrupts the T-wave of the preceding conducted beat
VF	A chaotic rhythm with undulations that are irregular in timing and morphology, without discrete QRS complexes on the surface ECG
ES	Three or more episodes of sustained VA occurring within 24 h, separated by at least 5 min, each requiring termination by an intervention
Clustered VAs	Two or more sustained VA events within 3 months

ECG, electrocardiogram; ES, electrical storm; PVC, premature ventricular complex; TdP, torsade de pointes; VA, ventricular arrhythmia; VF, ventricular fibrillation; VT, ventricular tachycardia.

The clinical presentation depends on multiple factors, including the underling cardiac disease, biventricular function, the type and CL of the VA, and the time to and mode of termination.^74^ Both cardiac function and non-cardiac comorbidities (including renal impairment, chronic pulmonary disease, and sepsis) influence the clinical presentation, electrical stability, and prognosis.^75–78^ Identification of patients at high risk and early transfer to a dedicated unit allowing for mechanical ventilation and mechanical circulatory support (MCS) is important.

Patients with ICDs, in whom the VA episode is terminated promptly by ATP, are likely to improve earlier than patients with prolonged VT episodes terminated by ICD shocks after ATP failure.^79^ Of note, even in patients with ICDs and good functional status, frequent episodes of slow, acutely tolerated and appropriately terminated VTs, can aggravate ventricular dysfunction and increase New York Heart Association (NYHA) class and cardiac mortality.^80^

Electrical storm can induce or exaggerate cardiac ischaemia, leading to cardiogenic shock and renal failure with metabolic acidosis and electrolyte disturbance, further promoting electrical instability.

## 8. Acute management

### 8.1. Initial evaluation, assessment of external precipitating factors, and reversible causes

Assessment of vital signs and the haemodynamic status (consciousness level, body temperature, heart rate, respiratory rate, and blood pressure), followed by continuous assessment of the rhythm and haemodynamic monitoring, is mandatory in patients presenting with an ES.^18,81,82^

#### 8.1.1. History

Collecting all information on the underlying heart disease and the patient’s functional status and comorbidities are essential. Thorough family history, including a history of sudden cardiac deaths or diagnosed PED, plays a critical role, especially in young patients with the first presentation. The drug history, including all prescribed and free available drugs, in particular, if recently initiated or up-titrated, may give a first suspicion of drug-induced arrhythmias [e.g. QRS or QT-prolonging drugs (http://www.crediblemeds.org) or drugs that unmask Type 1 Brugada pattern (https://www.brugadadrugs.org)], drug–drug interaction, or electrolyte abnormalities (e.g. thiazide and loop diuretics) that may contribute to electrical instability. Recent changes in the clinical status (e.g. chest pain, dyspnoea, new signs of cardiac decompensation, infection, or palpitations) or non-cardiac symptoms (vomiting, diarrhoea, and dehydration that can contribute to electrolyte abnormalities) should be noted. Factors that may be associated with an ES should be gathered from family members/accompanying persons in case of unconscious patients.^25^

#### 8.1.2. Twelve-lead electrocardiogram

**Figure 2:**
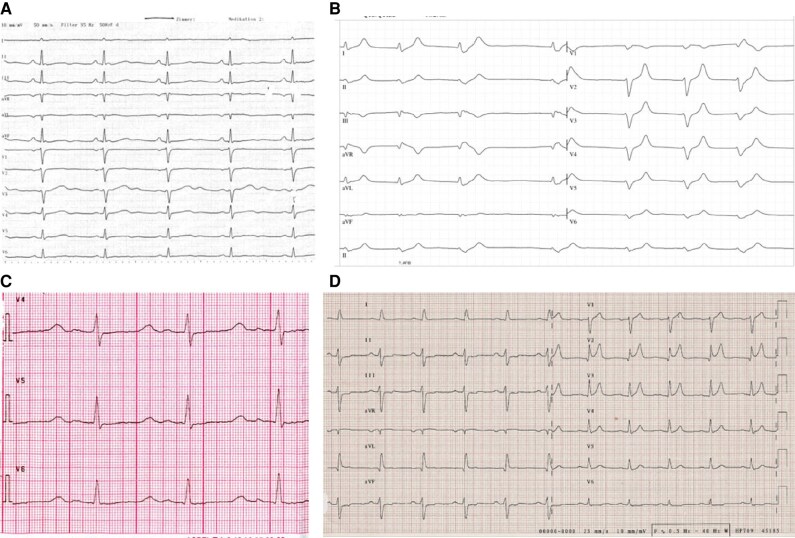
Manifestations of electrolyte-level disturbances on ECG: (*A*) hypokalaemia, (*B*) hyperkalaemia, (*C*) hypocalcaemia, (*D*) hypercalcaemia. ECG, electrocardiogram.

Recording the 12-lead electrocardiogram (ECG) after VA termination and, whenever possible, during the VA episodes is mandatory. The ECG can be diagnostic for ST-elevation MI (STEMI) but may also show signs of myocardial ischaemia, which may be the consequence of VA. Therefore, repeated ECGs are required to evaluate whether ECG changes consistent with ischaemia (e.g. ST depression) resolve shortly after VA termination or persist. The ECG after VA termination may also point towards precipitating factors (e.g. prolonged QT interval, Brugada pattern, signs of hypokalaemia) but may also indicate a yet undiagnosed underlying heart disease (*Tables 4* and *5* and *Figure 2*). The ECG of the VA can also contribute to the diagnosis of a potential underlying disease (e.g. bidirectional VT for CPVT or TdP for LQT) but may also provide important information on substrate location (*Table 6*).

**Table 4:** Baseline ECG changes suspicious for precipitation factors/underlying aetiologies

Baseline 12-lead ECG		Condition/diagnosis
PED	QTc ≥ 460 ms	LQTS (including acquired LQT)
	Type 1 Brugada ECG J-point elevation ≥2 mm with coved ST-elevation and T-wave inversion in at least one right precordial lead	Brugada syndrome Exclude other conditions (phenocopies)
	Early repolarization pattern J-point elevation ≥1 mm in ≥2 adjacent inferior and/or lateral ECG leads	Early repolarization syndrome
SHD	Pathological Q waves: >40 ms, >0.2 mV, >25% of QRS amplitude, any in V1–3 except LBBB	cCAD
	QRS fragmentation^a^	cCAD, SHD
	ST-segment elevation : J-point elevation ≥2 mm in V2/V3 in men, ≥1.5 mm in women, or ≥1 mm in 2 other contiguous chest leads or limb leads	STEMI
	Epsilon wave V1–V3: low amplitude signal occurring after the QRS complex and before the onset of the T wave	ARVC, cardiac sarcoidosis

^a^An additional R′, notching of the R wave, notching of the S wave, or the presence of multiple (>2) R waves, in the absence of a wide QRS.^65^

ARVC, arrhythmogenic right ventricular cardiomyopathy; cCAD, coronary artery disease; ECG, electrocardiogram; LBBB, left bundle branch block; LQTS, long QT syndrome; PED, primary electrical disease; QTc, corrected QT; SHD, structural heart disease; STEMI, ST-elevation MI.

**Table 5:** Electrolyte disturbances—ECG, causes and management

	Hypokalaemia	Hyperkalaemia
ECG findings	T-wave flattening/inversion Widespread ST depression Prominent U wave Increased P-wave amplitude Prolongation of PR interval Long QU interval	Peaked T waves P-wave widening/flattening PR prolongation QRS widening Bradyarrhythmias: sinus bradycardia, high-grade AV block with slow junctional and ventricular escape rhythms, slow AF Conduction blocks (bundle branch block, fascicular blocks)
Causes	Abnormal losses: medications (diuretics, laxatives, corticosteroids), gastrointestinal losses, renal losses, hypomagnesaemia, dialysis Transcellular shifts: medications: (insulin overdose, B2-sympaticomimetics, decongestants), alkalosis, thyrotoxicosis, hypothermia, head injury, myocardial ischaemia Inadequate intake: anorexia, dementia, parenteral nutrition	Increased intake: potassium supplementation, red blood cell transfusion Impaired excretion: kidney disease, congestive heart failure, cirrhosis, medications (potassium-sparing diuretics, ACE inhibitors, ARBs, heparin), hypoaldosteronism Transcellular shifts: insulin deficiency, acidosis, medications (BBs, digoxin toxicity) Pseudo-hyperkalaemia: haemolysis, leucocytosis (>75 000 cells per mm^3^), erythrocytosis, thrombocytosis
Management	Oral potassium chloride: 40–100 mmol i.v. 20–40 mmol/L potassium chloride in 500 mL of saline (not exceed 20 mmol/h; higher rates in emergency situation via central venous catheter)	Potassium <6 mEq/L: stop potassium-elevating drugs, 15–30 g sodium polystyrene sulfonate orally or rectally Potassium >6 mEq/L: insulin with glucose (5–10 unit with 50 mL 50% glucose), calcium chloride 10 mL of 10% solution i.v. over 5–10 min or calcium gluconate 30 mL of 10% solution i.v. over 5–10 min, beta 2-antagonist (salbutamol 0.25–0.5 mg iv, repeated dose after 15 min), dialysis

	Hypocalcaemia	Hypercalcaemia
ECG findings	QTc prolongation (ST-segment prolonged) The T-wave unchanged Dysrhythmias uncommon TdP may occur	Shortening of the QT interval Osborn waves (J waves) PVCs/PVT/VF
Causes	Hypoparathyroidism Pseudo-hypoparathyroidism Renal disease Vitamin D deficiency and dependency Acute pancreatitis Drugs: phenytoin, phenobarbital Infusion of gadolinium Magnesium depletion Septic shock	Primary hyperparathyroidism Cancers (lung, breast, multiple myeloma, renal cell carcinoma, prostate, ovarian, lymphoma, sarcoma) Drugs: hydrochlorothiazide, lithium, calcium supplements, vitamin D toxicity Sarcoidosis Renal disease Thyrotoxicosis
Management	i.v. calcium gluconate 10 mL of 10% solution over 10 min 1–2 g of oral calcium for post-operative hypoparathyroidism 1–2 g of oral calcium and vitamin D for chronic hypocalcaemia	Serum calcium <11.5 mg/dL (< 2.9 mmol/L): oral phosphate 250–500 mg 4 times a day serum calcium <18 mg/dL (<4.5 mmol/L): iv 500–1000 mL of saline and furosemide 20–40 mg every 2–4 h serum calcium 11.5 to 18 mg/dL (3.7 to 5.8 mmol/L) and/or moderate symptoms: iv 500–1000 mL of isotonic saline and furosemide 20–40 mg every 2–4 h, bisphosphonates (zoledronate 4–8 mg iv) or other calcium-lowering drugs serum calcium >18 mg/dL (>5.8 mmol/L): haemodialysis

	Hypomagnesaemia	Hypermagnesaemia
ECG findings	Prolonged QT interval Prolonged PR interval Atrial and ventricular ectopy VT and TdP	Flat P wave Prolonged PR interval Widened QRS complex Tall T wave
Causes	Gastrointestinal: reduced intake, reduced absorption, increased losses (diarrhoea, laxative abuse) Increase renal excretion: drug-induced (loop diuretics, thiazides, proton pump inhibitors), hypercalcaemic states, hyperaldosteronism	Impaired renal excretion: renal failure, hypothyroidism, adrenal insufficiency, lithium therapy Excessive intake: as treatment for peptic ulcer disease Excessive absorption: gastric or intestinal inflammatory
Management	Oral magnesium salts In severe cases [magnesium <1.25 mg/dL (<0.5 mmol/L)] and life-threatening arrhythmia: magnesium sulfate 1–2 g i.v. over 30–60 s and repeat in 5–15 min if necessary In asymptomatic patients and magnesium <1.25 mg/dL (<0.5 mmol/L): magnesium sulfate 1–2 g/h i.v. for up to 10 h	Ca2+ in as 10–20 mL of 10% calcium gluconate i.v. i.v. administration of 1000–2000 mL of 0.9% NaCl and furosemide 20–40 mg Haemodialysis using magnesium-free or low-magnesium dialysate

ACE, angiotensin-converting enzyme; AF, atrial firbrillation; ARB, angiotensin receptor blocker; AV, atrioventricular; BB, beta-blocker; ECG, electrocardiogram; PVC, premature ventricular complex; PVT, polymorphic ventricular tachycardia; TdP, torsade de pointes; VF, ventricular fibrillation; VT, ventricular tachycardia.

**Table 6:** Twelve-lead ECG during VT

ECG during VT	Description	Suggestive cause of ES/site of origin
TdP	PVT with ‘QRS twisting'	Acquired or congenital LQT
PVT	VT with continually changing QRS morphology	Acute ischaemia, primary electrical disease (BrS, SQT, IVF, CPVT, ERS)
Bidirectional VT	Alternating QRS morphology (beat-to-beat)	CPVT, digitalis intoxication, Andersen–Tawil syndrome
LBBB-like and inferior axis^a^		ARVC, idiopathic VT from the RVOT (normal heart)
RBBB-like VT with left superior axis (LAH-like, less frequently right inferior axis LPH-like^a^)^83^	Narrow QRS (100–140 ms), short RS interval (onset of R wave to nadir of S wave of 60–80 ms), r < R′ in V1	Left ventricular fascicular VT (usually normal heart)
	QRS broad (≥150 ms), absence of rsR′ in V1	Papillary muscle VT
	QRS broad (≥155 ms), R ≥ s in V5, may be positive QRS concordance	Peri-mitral VT
Fast (>200 b.p.m.) LBBB-like VT, with rapid downstroke of S wave in the anterior precordial leads, QRS during sinus rhythm (usually broad) may be identical to QRS during^a^ VT^84,85^	Short QRS onset—the R-wave peak time (R-wave peak time) and fast downstroke of S wave in V1–V2	Bundle branch re-entrant tachycardia (SHD)
RBBB-like VT with pseudo-delta wave and broad RS interval,^4^ absence of q waves in inferior leads and presence in Lead^a^ I^86–88^	Pseudo-delta wave ≥34 ms (interval from the earliest ventricular activation to the earliest fast deflection in any precordial lead) R-wave peak time in V2 ≥85 ms Shortest RS complex ≥121 ms (in any precordial lead)	VT with epicardial origin (SHD)

^a^LBBB-like pattern is characterized by a negative net amplitude of QRS complexes in leads V1 and/or V2 and RBBB-like by a positive net QRS amplitude in these leads.

ARVC, arrhythmogenic right ventricular cardiomyopathy; BrS, Brugada syndrome; CPVT, catecholaminergic polymorphic ventricular tachycardia; ECG, electrocardiogram; ES, electrical storm; ERS, early repolarization syndrome; IVF, idiopathic ventricular fibrillation; LAH, left anterior hemiblock; LBBB, left bundle branch block; LPH, left posterior hemiblock; LQT, long QT; PVT, polymorphic ventricular tachycardia; RBBB, right bundle branch block; RVOT, right ventricular outflow tract; SHD, structural heart disease; SQT, short QT; TdP, torsade de pointes; VT, ventricular tachycardia.

#### 8.1.3. Documentation of ventricular arrhythmia and immediate implantable cardioverter-defibrillator interrogation

Evaluation of the appropriateness, necessity, and effectiveness of ATP and ICD shocks is required in all patients with an ICD—(*Flowcharts 1 and 2*). Collecting the available information on the initiation and mode of termination of all VA episodes from 12-lead ECG, wearables, cardiac monitor rhythm stripes, tracings stored in automated external defibrillator, or intra-cardiac electrograms obtained from ICD interrogation is crucial for the acute and chronic management of ES.^50,56^ If PVC-induced VF is likely, continuous recording of the 12 lead ECG is helpful to capture the morphology and to determine the likely site of origin in case of unifocal PVCs and helps to mark sites just in case of electrophysiologic study and ablation.^89^

**Flowchart 1:**
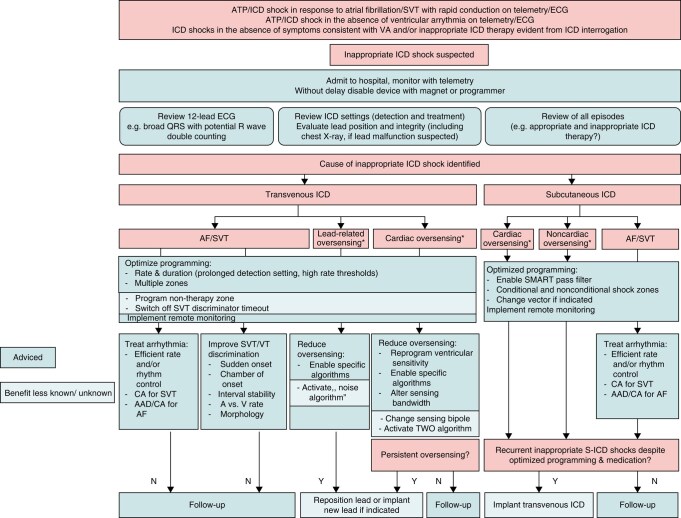
ICD interrogation and reprogramming—inappropriate therapy suspected.^139^*Cardiac oversensing: T-wave oversensing (most common cause of inappropriate shocks in S-ICD); P-wave oversensing; R-wave double counting. #Lead-related oversensing: conductor fracture; insulation breaches; connection problems; lead–lead interaction; air entrapment in the header. @Extracardiac oversensing: myopotentials from skeletal muscle activity (diaphragmatic, pectoral, intercostal rarely); electromagnetic interference (alternating current line breakdown, interference from medical sources). AAD, anti-arrhythmic drug; AF, atrial fibrillation; ATP, anti-tachycardia pacing; CA, catheter ablation; ECG, electrocardiogram; ICD, implantable cardioverter-defibrillator; SVT, supraventricular tachycardia; S-ICD, subcutaneous ICD; TWO, T-wave oversensing.

**Flowchart 2:**
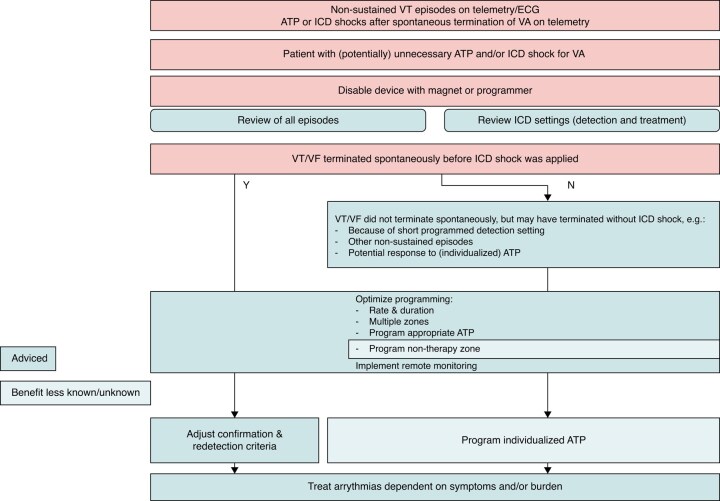
ICD interrogation and reprogramming—unnecessary therapy suspected.^139^ ICD, implantable cardioverter-defibrillator.

#### 8.1.4. Blood tests

Evaluation of serum electrolyte levels (Na^+^, K^+^, Mg^2+^, and Ca^2+^) is part of the initial evaluation.^25,72,73,90,91^ Hypomagnesaemia and/or hypokalaemia can be associated with TdP. Initial blood tests should also include thyroid-stimulating hormone to exclude hypothyroidism or hyperthyroidism as a potential trigger of ES (e.g. in amiodarone-induced thyrotoxicosis^92^). In addition, renal^77^ and liver function tests, impairment of which may lead to drug toxicity (e.g. sotalol), and blood tests for infection and inflammation may provide important diagnostic information on circumstances that require prompt treatment.

Initial lab testing should also include serum troponin, even though following ES, the diagnostic performance of cardiac injury markers alone is limited. In general, VA, in particular, if poorly haemodynamically tolerated, can cause myocardial injury and may lead to an increase in these markers.^93^ Therefore, troponin should be monitored sequentially and interpreted in the context of symptoms (angina pectoris), and ECG changes to identify and treat acute coronary syndrome (ACS). Toxicology screening for cocaine, alcohol, and other drugs may be appropriate in selected cases as abuse of those agents may lead to VA and even SCD.^94,95^ According to ESC guidelines, blood should be collected for potential later analysis.^1^

#### 8.1.5. Coronary angiogram, cardiac imaging, and additional diagnostic studies

Transthoracic echocardiography (TTE) should be performed early to assess cardiac function, dimensions, and disease progression in patients with known cardiac disease and to identify SHD in those without prior history of cardiac disease. Urgent coronary angiography (CAG) is essential in case of STEMI or electrical instability with suspicion of ongoing myocardial ischaemia.^96^ Of note, termination of incessant VT with intracoronary administration of radiographic contrast media, with subsequent haemodynamic stabilization, has been reported.^97^ If acute myocarditis is suspected, endomyocardial biopsy (EMB) is the gold standard for the diagnosis, which has important implications in cases of, e.g. giant cell myocarditis.^98^ 18F-fluorodeoxyglucose positron emission tomography can be useful in diagnosing arrhythmic myocarditis or cardiac sarcoidosis (CS), particularly when cardiac magnetic resonance (CMR) is contraindicated and is unsuitable because of irregular rhythm or ICD–related artefacts or when there is a diagnostic mismatch between CMR and EMB.^99,100^

**Advice Table 1:** Initial evaluation—general aspects

	Evidence	Strength
**Advice TO DO**		
Admit patients to a dedicated unit allowing multi-disciplinary patient management and continuous monitoring^18^	OBS	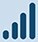
Assess vital signs and haemodynamic status, including conscious level, heart rate, respiratory rate, blood pressure, body temperature, and arterial haemoglobin oxygen saturation.	OPN	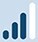
Investigate for external precipitating factors and reversible causes^77,90–92^	OBS	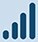
Collect data on current drug treatment and prior drug failure for VA	OPN	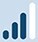
Record a 12-lead ECG of VA, whenever possible	OPN	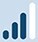
Record a 12-lead ECG after VA termination and repeat 12-lead ECG at least on a daily basis in selected cases (ST changes, QT prolongation)	OPN	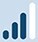
Perform TTE after VA termination For initial evaluation in patients without known disease For disease progression in patients with known structural cardiac disease	OPN	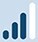
Evaluate appropriateness and necessity of ICD therapies^101–103^	OBS	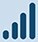
Reprogramme and/or inactivate ICD therapies in case of malfunction, inappropriate, or unnecessary therapies^104–106^	OBS	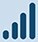
Collect all available information on type, initiation, and termination of all VA episodes, including telemetry strips and recordings from cardiac implantable electronic devices (CIEDs)^89,101^	OBS	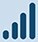
Perform urgent CAG in case of STEMI^107,108^ Persistent electrical instability and suspected ongoing ischaemia	OBS	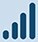
**May be appropriate TO DO**		
Initiate continuous 12-lead ECG monitoring in case of PVC-induced PVT/VF to determine similarity and likely site of origin of PVCs^89^	OBS	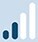
**Advice NOT TO DO**		
Do not perform routine toxicology screening	OPN	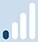

### 8.2. Treatment

#### 8.2.1. Electrical storm with haemodynamic instability

In case of haemodynamic instability, initiation of immediate ACLS is indicated.^1,109^ Unstable patients need to be managed by an interdisciplinary team, including a cardiologist with expertise in cardiac electrophysiology and cardiac implantable devices, an anaesthesiologist or intensive care specialist, and, in selected cases, a HF specialist or a cardiac surgeon. Patients may be haemodynamically unstable because of frequent episodes of VT or incessant VT with haemodynamic compromise. Haemodynamical instability may also persist after VT termination due to a delayed recovery after long episodes of VT and/or several ICD shocks or CVs, in particular in patients with poor cardiac function.

**Figure 3:**
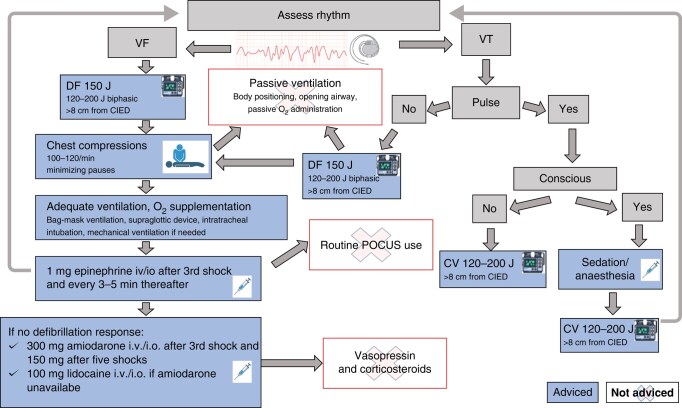
ACLS in an electrical storm.^110,113^ ACLS, advanced cardiovascular life support; CV, cardioversion; DF, defibrillation; POCUS, point of-care ultrasound.

Patients with ES and ongoing haemodynamically unstable VT or VF should be managed according to the general principles of ACLS (*Figure 3*).^110^ Basic airway techniques and a stepwise approach (bag-mask ventilation, supraglottic device or intratracheal intubation, and mechanical ventilation^75^) are advised until adequate ventilation is achieved. High-quality chest compressions with minimal interruption and early DF remain priorities. Rapid DF or CV may be delivered from the ICD or with an external defibrillator. When using external DF/CV in ICD patients, patches or paddles should be placed at least 8 cm from the ICD and, if possible, in a front-to-back position.^110^ External CV was shown to be similarly safe but superior to ICD shock for restoring sinus rhythm in patients with atrial fibrillation (AF) in a recent meta-analysis, but data on DF or CV for VT/VF are lacking.^111^ In a cluster-randomized trial including 405 patients with refractory VF, double sequential external defibrillation (DSED; rapid sequential shocks from two defibrillators) and vector change (VC) DF (switching DF pads to an anterior-posterior position) were associated with higher survival to hospital discharge and DSED (but not VC) with a better neurologic outcome.^112^ In patients with a non-shockable rhythm, adrenaline (1 mg i.v./i.o.) should be used immediately and repeated every 3–5 min during ACLS; in shockable rhythm, it should be administered after the third DF/CV and repeated every 3–5 min in case of persistent arrhythmia. Amiodarone (300 mg iv/io) is advised after three shocks in patients with VF/PVT, followed by 150 mg iv/io after five shocks. If amiodarone is unavailable, lidocaine (100 mg iv/io) can be alternatively used after three shocks and repeated (50 mg iv/io) after five DFs.

Conscious patients require deep sedation or anaesthesia before delivering synchronized CV (see Section 8.2.4.). Guidelines-recommended management of patients with VT and a palpable pulse advices a 120–150 J for the initial CV.^110^ The energy can be increased if the first shock fails.^110^ Even in haemodynamically tolerated VT, prompt termination, preferably by CV, is recommended by the current ESC guidelines, as haemodynamic deterioration may occur.^1^ Pharmacological treatment may be an alternative if the risk of sedation/anaesthesia is high (see Section 8.2.5.). Mechanical circulatory support may be appropriate in selected patients in whom conventional cardiopulmonary resuscitation (CPR) is not effective^114^ (see Section 8.2.6.). The role of therapeutic hypothermia in patients with ES is unknown.

**Advice Table 2:** Advanced life support

	Evidence	Strength
**Advice TO DO**		
Apply standardized advanced life support (ALS) algorithms including high-quality chest compression with minimal interruption^109,115^	RCT	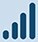
Use vasopressors (epinephrine) early for non-shockable cardiac arrest following ES^116–118^	RCT	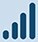
**May be appropriate TO DO**		
MCS may be appopriate when haemodynamical stabilization can not be achieved by conventional therapy^119^	OBS	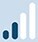

#### 8.2.2. Disease-specific aspects

Optimal HF management is mandatory and may help to stabilize the rhythm.^120,121^ In case of suspicion of ongoing myocardial ischaemia,^122^ urgent CAG is essential and is indicated in case of STEMI following current guidelines.^96^ In patients with cCAD but no STEMI, the potential benefit of (complete) revascularization should be carefully weighed against risk.^123,124^ Noteworthy, MSVT is rarely caused by ischaemia,^125^ and ischaemic evaluations in patients with monomorphic VT storm without ACS did not improve procedural outcomes or mortality after ablation.^126^

In the rare case of acute giant cell myocarditis, promptly initiated immunosuppression can be curative.^127^ In patients with CS and VA, corticoid therapy is advised by the ESC Working Group on Myocardial and Pericardial Diseases.^127^ Data on the acute effect of steroids in patients with active CS and ES are lacking, and AAD or RFCA may be needed.^128^ Anti-inflammatory therapy with corticosteroids or other immunosuppressive treatments may also be appropriate in myocarditis, if an autoimmune mechanism is suspected (circulating serum cardiac auto-antibodies and ongoing inflammation) and no active viral replication has been detected.^129^ In case of electrical instability in the inflammatory phase, AAD therapy and RFCA in case of drug-refractory storm may be appropriate. In a small cohort of patients with biopsy-proven chronic active myocarditis and drug-refractory VTs, RFCA effectively controlled MSVT.^130^

Acute illness and fever should be aggressively treated with fever-reducing medications, particularly in patients with BrS.^131^ Emotional stress should be considered as the potential trigger in patients with CPVT, and elevated sympathetic tone needs to be addressed in all patients with ES (see Sections 8.2.5.2. and 8.2.8.). Bradycardia may facilitate VA initiation, in particular in the setting of QT prolongation, and may require appropriate measures to increase heart rate (see Sections 8.2.3., 8.2.5.2., and 8.2.9.). Of note, the corrected QT (QTc)/corrected JT (JTc) interval can be significantly prolonged after resuscitation or in the setting of hypothermia, independently from QT-prolonging drugs, and normalization should be only expected beyond Day 6 after the acute event.^132,133^ Any potentially offending drug must be withdrawn, and drugs known to prolong the QT interval, except AADs used to treat the ES, must be avoided. Early correction of serum electrolyte levels (K^+^, Mg^2+^, and Ca^2+^) is indicated^134–136^; in cases of TdP, i.v. magnesium is effective even in the absence of hypomagnesaemia.^137^ I.v. potassium is usually effective, and K levels should be brought to the upper limits of normal range.

#### 8.2.3. Implantable cardioverter-defibrillator reprogramming

**Figure 4:**
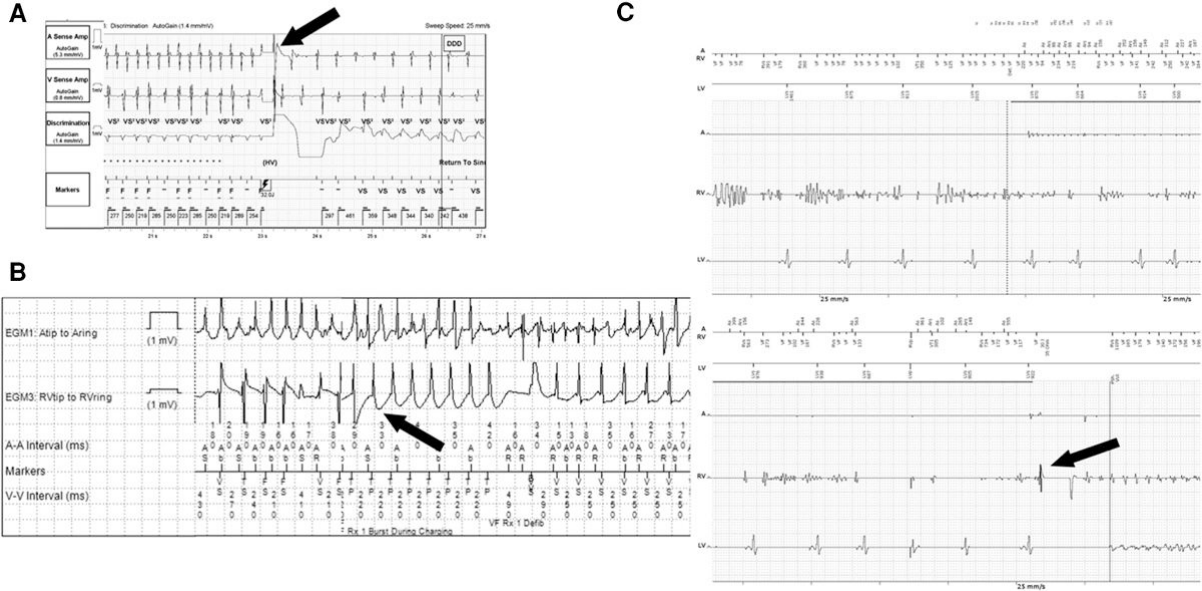
Inappropriate ICD therapy. (*A*) Fast conducting AF misdetected as VF with ICD shock (arrow), (*B*) fast conducting AF misdetected as VT with ATP delivered (arrow), (*C*) noise caused by damaged RV electrode misdetected as VF with ICD shock (arrow). Shock initiates VF. A, atrial signal; AF, atrial fibrillation; Aring, ring pole of atrial electrode; Atip, tip pole of atrial electrode; ATP, anti-tachycardia pacing; CRT, cardiac resynchronization therapy; EGM, intra-cardiac electrogram; F, ventricular fibrillation detected; ICD, implantable cardioverter-defibrillator; LV, signal received by electrode in left ventricle in CRT device; RV, signal received by electrode in the right ventricle; RVring, ring pole of electrode in the right ventricle; RVtip, tip pole of electrode in the right ventricle; STIM, stimulation; VF, ventricular fibrillation; VS or RVs, ventricular sensed event; VT, ventricular tachycardia.

Inappropriate ICD therapy can be due to supraventricular tachycardias or AF with a rapid ventricular response (*Figure 4*). Inappropriate ICD therapy can also result from cardiac or extracardiac oversensing or lead defects. Unnecessary ICD therapy may be triggered by non-sustained VTs, particularly if short detection times are programmed. In these cases, ICD therapies must be interrupted and prevented by disabling and reprogramming the device.^104^ If there is no immediate access to a device programmer and ICD malfunction or inappropriate ICD treatment is suspected from the telemetry or ECG recordings, ICD therapy can be transiently disabled by placing a magnet on the device can (although this is a manufacturer and model-specific feature, which can be programmable).

Recurrent, appropriate ICD shocks may not only be due to ineffective ATP or CV/DF and acceleration of VT by ATP (e.g. RAMP protocol set as a first mode) but also be due to programmed low VF detection rates, in particular if ATP during charging is not available or disabled.^104^ Programming of longer detection times and longer and less aggressive ATP therapies may reduce or avoid ICD shocks. In particular, in patients with haemodynamically well-tolerated VT, terminated by ICD shocks, disabling the device therapy and termination of episodes by commanded ATP or AADs are advised. Recurrent ICD shocks are traumatic events associated with increased mortality, and the related hyperadrenergic state may trigger recurrent episodes. Accordingly, patient sedation (see Section 8.2.4.) and inactivation of unnecessary ICD shocks, even if appropriate, may mitigate the ES.^105,106^

In addition, and depending on the mode of VA initiation, increasing the lower rate thresholds for anti-bradycardic pacing to avoid bradycardia and short-long-short sequences may prevent VA episodes^101^—(see Flowcharts 1 and 2; Section 8.2.9.). Although adequate LV or biventricular pacing of CRT is desirable, recently initiated effective LV stimulation may be arrhythmogenic, and disabling LV pacing may sometimes terminate or mitigate the ES.^138^

#### 8.2.4. Deep sedation and mechanical ventilation

In conscious patients with recurrent ICD shocks or symptomatic VTs, mild-to-moderate sedation is essential for patient comfort and to reduce sympathetic tone (*Table 7*). Deep sedation and mechanical ventilation have successfully controlled drug-refractory ES.^140,141^ An acute response, defined as the termination of ES within 15 min without recurrent episodes over the following 24 h, occurred in 47% of a mixed cohort of 116 ES patients who were refractory to AADs. Response to deep sedation was an independent predictor of in-hospital survival.^141^

**Table 7:** Medication for sedation in ES patients

Sedation^90–92^		
Agents	Initial dose	Infusion rate
Benzodiazepines		
Midazolam	0.01–0.05 mg/kg, repeat q5–15 min	0.02–0.1 mg/kg/h, titrate up/down by 25–50%
Lorazepam	0.02–0.04 mg/kg	0.01–0.1 mg/kg/h (not exceed 10 mg/h)
Propofol	0.8–1.2 mg/kg	5–50 μg/kg/min (0.3–3 mg/kg/h) Up-titrate every 5–10 min by 5–10 μg/kg/h
Opioids		
Fentanyl	1–2 μg/kg	1–2 μg/kg/h
Remifentanyl	0.5–1.5 μg/kg	0.05–2 μg/kg/min
Dexmedetomidine	1 μg/kg over 10 min	0.2–0.7 μg/kg/h

ES, electrical storm.

##### 8.2.4.1. Agents used for (deep) sedation

*Benzodiazepines*. Short-acting agents like midazolam are commonly used in patients with an ES. With no negative inotropic effect, benzodiazepines are considered first-line sedation drugs, often used for initial mild sedation.^140,142^

*Propofol*. Propofol is a potent intravenous hypnotic agent used for deep sedation in the context of an ES.^140,143^ Propofol may substantially inhibit sympathetic nerve activity; ^144^ it has a rapid onset of action within 2–3 min, a short half-life of 1–3 h, and good amnestic potential. This agent must be used cautiously in patients with SHD because of its negative inotropic effect.

In a retrospective study of 46 subjects presenting with VT storm or incessant VT, 15 patients refractory to AAD underwent propofol-induced deep sedation. After propofol administration, a complete resolution of VT/VF within minutes to hours was achieved in 12 patients (80%), and partial resolution in 2 (13%), with no VA recurrence after 3 h.^140^ Hypotension was the most common side effect managed primarily with norepinephrine infusion. One case of necrosis of the caecum was attributed to the complications of norepinephrine. Uncommon complications are hypertriglyceridaemia, pancreatitis, allergic reactions, and propofol-related infusion syndrome, which is an often lethal complication of high-dose propofol infusions, characterized by severe metabolic acidosis, bradyarrhythmias, acute renal failure, rhabdomyolysis, and hyperkalaemia.^145^ Propofol may occasionally significantly prolong QT interval.^146^

*Opioids*. Short-acting opioid agents are also considered first-line treatment because of their mild negative inotropic and potent analgesic effects.^74,147^ Fentanyl/remifentanil are used for the induction of general anaesthesia. The onset of action of fentanyl is <2 min, with a peak of 2–5 min after administration. The half-life is 90 min, and the duration of action is 30–60 min. Remifentanil has a distribution half-life of 2–4 min and elimination half-time of 10–20 min. Its offset of action (3–5 min) is not affected by the duration of drug infusion (no accumulation with repeated and prolonged administration), and its pharmacokinetics is not affected by renal nor hepatic impairment.

*Dexmedetomidine*. Dexmedetomidine is a highly selective α2-adrenoreceptor agonist with a short half-life (6 min) that reduces sympathetic activity by enhancing central vagal tone and inhibiting presynaptic catecholamine release. It provides both sedation and analgesia without respiratory depression and was shown to decrease the incidence of VT in critically ill patients.^148^ However, it should be used with caution as it may cause severe hypotension and bradycardia.

#### 8.2.5. Pharmacotherapy for the acute management of electrical storm

##### 8.2.5.1. Structural heart disease

Anti-arrhythmic drugs are the cornerstone of therapy for the acute management of ES (*Table 8*). The specific AAD treatment depends on the type of VA, the (likely) underlying aetiology, patient’s characteristics, including comorbidities and patient-specific contraindications, and the availability of drugs. In patients with SHD, suppressing sympathetic tone with beta-blockers (BBs), preferably non-selective (e.g. propranolol), is the first-line treatment unless contraindicated.^26^ In a randomized controlled trial (RCT), the combination of i.v. amiodarone and oral propranolol was safe, effective, and superior to the combination of i.v. amiodarone and oral metoprolol in the management of ES in ICD patients.^149^ In patients with an ES due to VT/VF after a recent (<3 months) MI, sympathetic blockade with i.v. esmolol or i.v. propranolol (160–320 mg/24 h) or left stellate ganglion blockade was superior to lidocaine i.v (followed by procainamide i.v. or bretylium, if not effective) for VT/VF suppression and survival.^26^ In patients with recurrent PVT in the early post-MI phase [but also post-coronary artery bypass grafting (CABG) and percutaneous coronary intervention (PCI)], quinidine has been reported to be effective after BB and amiodarone treatment failure.^150,151^ In patients with recurrent, haemodynamically unstable VT despite amiodarone, landiolol (ultra-short-acting β1-selective blocker) was effective for arrhythmia suppression in two smaller studies.^152,153^

**Table 8:** Pharmacotherapy for acute treatment of patients with ES

Acute treatment										
Vaughan Williams Class	Drug	Channels affected	Dose (i.v.)	Pharmacokinetics			Specific use	Caution	Monitoring	Adverse effects
				Half-life	Desired plasma concentration	Metabolism				
Class I	Quinidine	I_Na_, I_Kr_, I_To_, M, α	Loading dose: 800 mg/50 mL Maintenance intravenous: 50 μg/min	6–8 h	1.5–3.5 μg/mL	Hepatic	VT, VF, ES after MI, PCI, CABG, BrS, SQT syndrome IVF	Severe bradycardia and/or high-degree AV block in the absence of a pacemaker, myasthenia gravis, decompensated heart failure	ECG (QRS duration and QT interval), heart rate, platelet count	QTc prolongation, TdP, QRS prolongation, increase in defibrillation threshold, hypotension, bradycardia, heart failure exacerbation, diarrhoea, immune thrombocytopenia
	Procainamide	I_Na_, I_Kr_ ganglionic block	100 mg bolus, can be repeated after 5 min if no effect, alternatively 10–17 mg/kg administered at a rate of 20–50 mg/min, max 500–750 mg (max 50 mg/min) and then 2–6 mg/min	3–4 h	4–12 μg/mL	Hepatic and renal	VT, pre-excited AF	Severe sinus node disease, severe AV conduction disturbance, previous myocardial infarction, reduced LVEF, hypotension, BrS, myasthenia gravis	ECG (QRS duration and QT interval), blood pressure	Rash, myalgia, vasculitis, agranulocytosis, hypotension, bradycardia, QT prolongation
	Lidocaine	I_Na_	50–200 mg bolus and then 2–4 mg/min (25–50 μg/kg/min)	7–30 min	2–6 μg/mL	Hepatic	Ischaemic VT/VF	Severe sinus node/AV heart block in the absence of pacemaker, cardiogenic shock, hypersensitivity to lidocaine or amide-type local anaesthetist	ECG (PR interval and QRS duration), temperature	Hypotension, confusion, tremors, methemoglobinaemia, malignant hyperthermia, anaphylactoid reactions
	Mexiletine	I_Na_	Intravenous: not recommended Loading dose: 400 mg initially followed by 600 mg in the first 24 h Maintenance dose: 600–1200 mg	10–14 h	0.6–1.7 mg/mL	Hepatic	VT/VF, LQT3 LQT2	Cardiogenic shock, pre-existing sinus node disease, or second/third-degree AV block without pacemaker, hypotension, history of seizures	ECG (QT interval), hepatic function	Ataxia, tremors, hypotension, angina
Class II (BBs)	Esmolol	β1-receptor	Bolus dose: 0.5 mg/kg for 1 min Infusion: 25–50 μg /kg/min up to 250 μg /kg/min (titrate every 5–10 min)	5–10 min	NA	RBC esterase	VT	Severe sinus bradycardia/severe sinus node disease/AV conduction disturbances without pacemaker, Decompensated heart failure, Prinzmetal’s angina, asthma/chronic obstructive airway disease, myasthenia gravis	Heart rate, blood pressure	Bronchospasm, hypotension, sinus bradycardia, AV block, fatigue, depression
	Propranolol	Non-selective BB	Bolus dose: 0.15 mg/kg over 10 min, 160 mg/24 h	3–6 h	NA	Hepatic	VT, PVCs, LQT			
	Metoprolol	β1-receptor	Bolus dose: 2–5 mg every 5 min, up to 3 doses in 15 min	Tartarate: 3–4 h Succinate: 3–7 h	NA	Hepatic	VT, PVCs			
Class III	Amiodarone	I_Na_, I_Ca,_ I_Kr_, I_K1_, I_Ks_, I_to_, α, β	Loading dose: 5 mg/kg in 20 min to 2 h, 2–3 times in 24 h and then 600–1200 mg/ 24 h 8–10 days	4–14 weeks	1–2.5 μg/mL	Hepatic	VT, VF, PVCs	Concomitant digoxin administration, concomitant warfarin administration	Heart rate, blood pressure	Increases DFT, hypotension, bradycardia, AV block, QT prolongation, TdP (rare), hypothyroidism/hyperthyroidism, nausea, photosensitivity, skin discolouration, peripheral neuropathy, tremor, hepatitis, pulmonary fibrosis/pneumonitis
Beta agonist	Isoproterenol	β1 and β2	0.5–10 μg/min	2.5–5 min	NA	Hepatic and pulmonary	IVF TdP, ES in Brugada syndrome, SQT syndrome, VAs secondary to AV block	Coronary artery disease, myocardial ischaemia convulsions, renal disease, hyperthyroidism	Heart rate, blood pressure, ECG for ST-elevation	Tachycardia, hypertension, angina, tremors

AF, atrial fibrillation; BB, beta-blocker; BrS, Brugada syndrome; CABG, coronary artery bypass grafting; DFT, defibrillation threshold testing; ECG, electrocardiogram; ES, electrical storm; IVF, idiopathic ventricular fibrillation; LVEF, left ventricular ejection fraction; LQT, long QT; MI, myocardial infarction; NA, not applicable; PCI, percutaneous coronary intervention; PVC, premature ventricular complex; QTc, corrected QT; RBC, red blood cell; SQT, short QT; TdP, torsade de pointes; VF, ventricular fibrillation; VA, ventricular arrhythmia; VT, ventricular tachycardia.

Amiodarone i.v. (5 mg/kg/20 min) is frequently used in patients with SHD who present with an ES. It terminated haemodynamically tolerated VT in 38% of patients, the majority (80%) with SHD.^154^ Effects of oral vs. intravenous loading with amiodarone may differ; combined i.v. and oral loading may shorten the time to VA control compared with oral loading alone and may lower the cumulative dose required.^155^ Patients already on amiodarone therapy may benefit from acute reloading with this AAD.^142^

In patients with SHD and haemodynamically tolerated VT without acute ischaemia, procainamide can also be used. In the PROCAMIO trial, procainamide was superior to amiodarone in terminating haemodynamically tolerated VT of unknown aetiology, with fewer major adverse events.^154^ Lidocaine is only moderately effective (and worse than procainamide i.v.^156^ and sotalol i.v.^157^) in stable MSVT but has been considered potentially useful in the context of acute ischaemia (bolus 1–1.5 mg/kg followed by infusion rate of 0.02 mg/kg/min).^158^ Its prophylactic use had little or no effect on mortality or VF in AMI patients in a meta-analysis of controlled RCTs.^159^ As negative inotropic effects of lidocaine have been demonstrated in animal studies,^160^ the drug needs to be used with caution in patients with impaired ventricular function. Ranolazine, an antianginal and anti-ischaemic drug, also exerts anti-arrhythmic properties, presumably due to the inhibition of the late sodium and the delayed rectifier potassium current.^161^ In a double-blind RCT including 1012 ICD patients with SHD (54% with cCAD), ranolazine significantly reduced the risk of recurrent VT or VF requiring ICD therapy (pre-specified secondary endpoint) without an impact on mortality during a mean follow-up of 28 ± 16 months.^162^ The use of other AADs depends on the underlying aetiology and the type of VA.^163^

##### 8.2.5.2. Primary electrical disease

In patients with PED, treatment of an ES is disease specific. In LQTS, it involves the immediate elimination of eventual triggers [acquired LQTS (aLQTS) and congenital LQTS (cLQTS)], BBs, ideally non-selective (nadolol or propranolol), intravenous extracellular magnesium and potassium supplementation (aLQTS and cLQTS),^134,135,137^ isoproterenol (aLQTS), mexiletine (cLQTS), and temporary pacemaker therapy with supra-normal rates (aLQTS and cLQTS Type 2).^3^ Class III AADs should be avoided.

In BrS and IVF, an ES is best treated with isoproterenol i.v.^164–166^ Quinidine with or without isoproterenol has also been used successfully in individual cases.^167–172^ Class Ic drugs should be avoided. Short QT syndrome is very rare, and data regarding the treatment of ES are very limited; isoproterenol may be efficient in such cases.^173^

In CPVT, ES is rare and almost exclusively triggered by adrenergic stressors (including erroneous administration of epinephrine during resuscitation efforts). Treatment should therefore be directed towards the radical removal of all adrenergic triggers. Beta-blockers remain the first line of therapy, and epinephrine should be avoided, including in the setting of cardiac arrest/resuscitation.^174^

In all inherited arrhythmic syndromes or arrhythmogenic cardiomyopathies, where adrenergic stress is causally involved, the ultimate therapeutic option is deep anaesthesia (see Section 8.2.4.).

#### 8.2.6. Mechanical circulatory support

Temporary MCS may be beneficial in treating life-threatening, refractory, haemodynamically non-tolerated VA, providing maintenance of organ perfusion while allowing for the treatment of the precipitating arrhythmia. However, MCS is associated with non-negligible rates of complications (vascular access-related, bleeding, etc.), procedural logistics/complexity/availability, and increased costs. The lack of robust evidence on MCS use in the setting of ES makes it difficult to draw any definitive conclusions about its role in this scenario.

**Figure 5:**
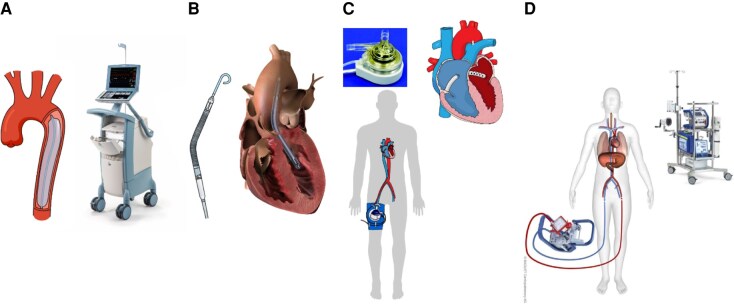
Types of mechanical circulatory support: (*A*) intra-aortic balloon pump, (*B*) transvalvular microaxial flow pump (Impella), (*C*) tandem heart, (*D*) veno-arterial extracorporeal membrane oxygenator.

Current temporary modalities for MCS include the intra-aortic balloon pump (IABP), the Impella devices (Abiomed, Danvers, MA, USA), Tandem Heart left atrial-to-femoral bypass (Cardiac Assist Inc., Pittsburgh, PA, USA), extracorporeal membrane oxygenation (ECMO), and peripheral cardiopulmonary bypass. Their main characteristics are depicted in *Table 9* and *Figure 5*. Mechanical circulatory support device selection depends on the patient’s characteristics, required support level, local expertise, and logistics. Notably, the use of temporary MCS is highly complex and should only be performed by experienced multi-disciplinary teams in specialized centres. The role of temporary MCS in ES has not been extensively investigated. Mechanical circulatory support may be helpful in patients presenting with ES in the following scenarios:

**Table 9:** Types of mechanical cardiac support

			Pros	Cons	Contraindications
IABP	Indirect LV support by aortic counterpulsation, with afterload reduction and diastolic aortic pressure augmentation	Percutaneous arterial access (7–8 Fr) Modest cardiac output increase (0.5–1.0 L/min)	Simple set-up Indirectly unloads LV Availability Affordable Low complication rate	Very limited LV support Requires stable rhythm	Moderate-severe aortic regurgitation Severe peripheral artery disease Aortic dissection
Impella CP	Microaxial continuous anterograde flow pump LV aorta	Maximum average flow 3.7 L/min Percutaneous arterial access (femoral/axillary) Introducer diameter 14 Fr Pump motor 14 Fr	Relatively simple set-up and implantation Unloads LV Availability Percutaneous removal	Partial LV support Electromagnetic interference in mapping system. Difficult catheter manipulation during ablation May require transeptal access for CA High costs	Moderate-severe aortic regurgitation Mechanic aortic prosthesis LV thrombus Severe peripheral artery disease Severe RV failure
Impella 5.5	Microaxial continuous anterograde flow pump LV aorta	Maximum average flow 5.5 L/min Surgical axillary cut-down Introducer diameter 23 Fr Pump motor 19 Fr	Full LV support Unloads LV Prolonged support duration Allows early mobilization	Requires surgical team/complex logistics Surgical insertion and removal High costs	Moderate-severe aortic regurgitation Mechanic aortic prosthesis LV thrombus Severe peripheral artery disease Severe RV failure
Impella RP	Type of MCS	Main characteristics	Technical details	Anecdotical experience in ES or VT ablation Only available in selected sites High costs	Mechanical valves, severe valvular stenosis/regurgitation of the tricuspid or pulmonary valve Right atrium or vena cava thrombus Presence of a vena cava filter or caval interruption device
TandemHeart	Left atrial-to-femoral artery bypass using external centrifugal pump	Percutaneous or surgical access Transseptal puncture required Generated output up to 5 L/min Inflow cannula 21 Fr Outflow cannula 15 Fr	LV support Indirectly unloads LV Prolonged support duration	Complex logistics. Multi-disciplinary team May impede transeptal approach for CA Large bore cannulas	Moderate-severe aortic regurgitation RV failure Ventricular septal defect Severe peripheral artery disease
VA-ECMO	Cardiopulmonary support system integrated by a centrifugal pump and an oxygenation membrane	Percutaneous/surgical vascular access 17–21 Fr outflow cannula 19–25 Fr inflow cannula Maximum output up to 8 L/min	Full biventricular support Prolonged support duration Default choice in isolated RV failure May be percutaneously removed	Complex logistics. Multi-disciplinary team Requires often arterial distal perfusion cannula to prevent leg ischaemia High complication rates	Severe aortic regurgitation Aortic dissection Severe peripheral artery disease (in peripheral cannulation) Uncontrollable bleeding or contraindications to systemic anticoagulation

ES, electrical storm; IABP, intra-aortic balloon pump; LV, left ventricle; MCS, mechanical circulatory support; RV, right ventricle; VA-ECMO, veno-arterial extracorporeal membrane oxygenation; VT, ventricular tachycardia

##### 8.2.6.1. Rescue therapy in acute haemodynamic decompensation because of refractory, haemodynamically intolerant ventricular arrhythmia

Because of the level of support provided and its rapid instauration, veno-arterial ECMO is the most attractive option for patients presenting with haemodynamic deterioration due to VA (ES, VT refractory to AADs, recurrent VF, or refractory cardiogenic shock after VA cessation).^18^ In an RCT in patients with incessant shockable rhythm (VT/VF), extracorporeal CPR (ECPR) was associated with lower mortality to hospital discharge compared with conventional CPR (7 vs. 43%).^175^ On the other hand, in another RCT including 256 patients with out-of-hospital cardiac arrest (OHCA) of presumably cardiac cause (61% with VF as presenting rhythm), ECPR failed to show a significant benefit in mortality when compared with conventional CPR (32 vs. 22%, *P* = 0.09).^176^ In a recent multi-centre RCT on 160 patients with OHCA due to refractory VT/VF, ECPR was not superior to conventional CPR in terms of mortality at 30 days (20 vs. 16%, *P* = 0.52).^177^ Systematic selection of optimal candidates and refinement of patient care through the process remain the cornerstone for a successful ECPR programme. The use of other MCS devices in this context, such as Impella, is anecdotal and only supported by small case series.

##### 8.2.6.2. Rescue therapy in peri-procedural acute haemodynamic decompensation because of refractory unstable ventricular arrhythmia in patients undergoing ventricular tachycardia ablation

Acute haemodynamic deterioration during VT ablation may occur in up to 11% of patients and is associated with increased mortality, both in the short and long term.^178^ Veno-arterial ECMO is the preferred rescue MCS modality in patients with ES undergoing CA. However, reported mortality rates in the short-term are high, ranging from 38 to 76%, despite successful CA.^179,180^ Of note, rescue MCS during CA was associated with a higher mortality rate than pre-emptive MCS or no-MCS.^181^

##### 8.2.6.3. Prophylactical mechanical circulatory support implantation prior to ventricular tachycardia ablation in patients at high risk of developing haemodynamic instability

Clinical evidence is scarce despite the potentially rational arguments for adopting this strategy. The selection of patients is the cornerstone of the strategy. The PAINESD score (*Table 10*) may help to estimate the risk of peri-procedural haemodynamic decompensation in patients with scar-related VTs requiring ablation.^119,178^ The risk of haemodynamic deterioration during the procedure may be influenced by the mapping strategy, in particular if repeated VT induction or mapping during VT is deemed necessary. In patients with a high PAINESD score (≥ 15), prophylactic MCS may reduce mortality.^182,183^ Prophylactic use of MCS was associated with survival benefits, as shown by a meta-analysis of 2465 patients with ES or high PAINESD scores.^119^ However, MCS as a support for ablation was associated with higher complication risk and procedural time/fluoroscopy burden.^184^ The vascular access strategy, the need for LV venting, and the expected duration of support time are mandatory to consider before the procedure.

**Table 10:** PAINESD scores

Parameter	Points
Pulmonary disease (COPD)	5
Age > 60 years	3
IHD	6
NYHA Class III and IV	6
EF < 25%	3
Storm electrical	5
DM	3
Low risk: ≤8, median risk: 9–14, high risk: ≥15 points	

COPD, chronic obstructive pulmonary disease; DM, diabetes; EF, ejection fraction; IHD, ischaemic heart disease; NYHA, New York Heart Association;

**Advice Table 3:** Mechanical circulatory support

	Evidence	Strength
**May be appropriate TO DO**		
MCS may be appropriate in patients with acute ES for optimizing haemodynamics^18,175–177^	RCT	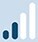
MCS may be appropriate in patients undergoing ablation of non-tolerated VAs to support mapping and ablation^179,180^	OBS	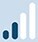
MCS if started before ablation may be extended to aid elective CA	OPN	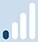
Initiation of MCS prophylactically before CA may be appropriate in high-risk patients, presenting with an ES or clustered VA^119,181,182^	OBS	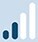

#### 8.2.7. Acute catheter ablation

Catheter ablation is the treatment of choice for monomorphic VT refractory to drug therapy in the acute phase and is advised in all eligible patients to reduce ES recurrence.^1,185^

In haemodynamically stable patients, expedited CA may be appropriate if non-invasive strategies cannot achieve prompt rhythm control. Catheter ablation aims to suppress VA and to prevent prolonged low-output state that can lead to acute multi-organ failure. Consideration of early CA is reasonable in patients with incessant VA or recurrent episodes of PVC-induced VA (also PVTs) that are haemodynamically significant despite AAD and deep sedation.

The ideal timing for CA remains unclear. However, in a recent meta-analysis, stabilization of patients with an ES using elective MCS prior to CA of VT appeared to be beneficial, compared with urgent MCS or non-MCS (see Section 8.2.6.).^119^ Rescue CAs within the first 24 h after ES onset with ongoing cardiogenic shock due to sustained VA episodes are associated with a higher rate of ablation failure and VA/ES recurrence.^186^ Evidence also suggested that CA performed after the first 48 h from admission is associated with lower mortality at 30 days compared with conservative therapy or very early ablation.^187^ Therefore, haemodynamic stabilization and arrhythmia suppression before proceeding with CA should be attempted but may not always be possible.

Catheter ablation in patients presenting with ES and PVT/VFs can be challenging. Trigger elimination is an effective strategy to achieve short-term and long-term ES freedom in post-MI patients with focally triggered VF storm (see Sections 8.1. and 8.2.2.).^188–190^ Epicardial approach may be needed in refractory cases in NICM, but also in cCAD patients.^191–193^ Due to the complex nature of VA ablations in ES that often require MCS, epicardial access, skilled operators, and support of multi-disciplinary staff, performing these ablations in experienced centres after initial stabilization is advised.

Urgent heart transplantation may be an option for selected patients who have a VA storm refractory to the therapy.

**Advice Table 4:** Acute treatment of ES in SHD

	Evidence	Strength
**Advice TO DO**		
Design a management plan for each ES patient in a multi-disciplinary team	OPN	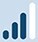
Start i.v. amiodarone (in combination with propranolol) in patients with SHD and reduced systolic function^149^	RCT	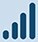
Quinidine is appropriate in patients with recurrent PVT resistant to amiodarone and BBs, in the early post-MI phase or post-coronary revascularization intervention^150,151^	OBS	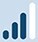
Perform VT ablation in patients with recurrent MSVT despite optimal medical therapy, provided that an experienced team is available^14,186^	OBS	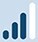
Perform CA in patients with recurrent episodes of VF triggered by a similar PVC despite optimal medical therapy, provided that an experienced team is available^189,190^	OBS	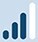
Transfer patients with recurrent MSVT or recurrent episodes of VF triggered by a similar PVC despite optimal medical therapy to a specialized centre for VA ablation, whenever possible^18^	OBS	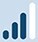
**Advice NOT TO DO**		
Do not apply invasive treatment in case of specific directive or ‘do not resuscitate’ orders	OPN	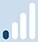

**Advice Table 5:** Acute treatment of unstable ES in PED

	Evidence	Strength
**Advice TO DO**		
Isoproterenol infusion is beneficial in BrS patients presenting with an ES^164,166–169,172^	OBS	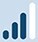
Intravenous magnesium and potassium supplementation is useful in the acute phase of LQTS-associated TdP. Remove or correct external triggers (i.e. drugs, electrolyte imbalance).^134,135,137^	OBS	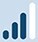
**May be appropriate TO DO**		
Isoproterenol or quinidine may be clinically useful to acutely treat an ES in patients with idiopathic VF^194–196^	OBS	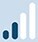
Isoproterenol infusion may be appropriate in ERS patients presenting with ES^197^	OBS	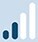
Isoproterenol may be appropriate in SQTS patients with an ES^173^	OPN	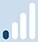
Verapamil may be clinically useful to acutely treat an ES in patients with idiopathic VF^195^	OPN	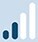
**Advice NOT TO DO**		
Catecholamines such as epinephrine and isoproterenol are not appropriate in CPVT patients with an ES^174^	OBS	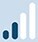

#### 8.2.8. Autonomic modulation

The autonomic nervous system plays an important role in the genesis of VT/VF.^198–201^ (see Section 6.4.). Beta-blocker therapy can decrease the risk of sudden cardiac death.^202^ Specifically, in the setting of ES, propranolol has demonstrated better efficacy compared with metoprolol.^149^ Of note, the effects of sympathetic activation extend beyond those influenced by BB therapy. Elevated sympathetic tone also leads to the release of sympathetic cotransmitters, such as neuropeptide Y, that likely decrease APD and influence arrhythmogenesis.^203,204^

In addition to increasing sympathetic efferent tone, sympathetic afferent activation may also play a role by inhibiting vagal tone. Therefore, sympathetic neuromodulatory therapies, such as stellate ganglion blockade (SGB), thoracic epidural anaesthesia (TEA), cardiac sympathetic denervation (CSD), and renal denervation (RDN), by changing efferent and afferent sympathetic signalling of the heart (afferent renal and efferent heart signalling in case of RDN), address mechanisms beyond blockade of beta-adrenergic receptors.

##### 8.2.8.1. Stellate ganglion block

**Figure 6:**
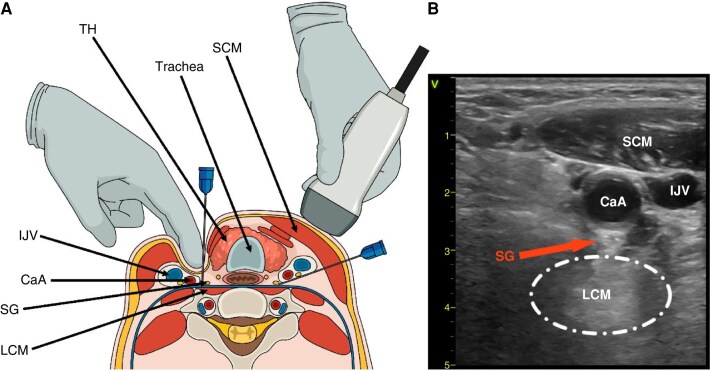
Stellate ganglion block. (*A*) Neck cross-section showing SGB guided by surface landmark technique and palpation (left) and ultrasound (right). (*B*) Transverse sonographic view of the neck at the level of sixth cervical vertebrae (C6). CaA, carotid artery; IJV, internal jugular vein; LCM, longus colli muscle; SCM, sternocleidomastoid muscle; SG, stellate ganglion; SGB, SG blockade; TH, thyroid.

Stellate ganglion block is performed with an anaesthetic agent (bupivacaine or ropivacaine) at the left or bilateral stellate ganglia level under ultrasound and/or fluoroscopic guidance (*Figure 6*).^205,206^ In several case series, pharmacological SGB reduced VT burden in patients with refractory VT or VT storm and was more effective than standard ACLS protocol using amiodarone, though prospective and randomized data are needed.^26,205–208^ Continuous infusion of anaesthetic agents using a catheter placed near the stellate ganglion can provide better control of VA than a single bolus injection.^209^ Of note, efficacy of cryoenergy-induced and radiofrequency-induced SGBs have been reported in small case series.^210,211^ In a recent RCT in 26 patients with refractory ES, transcutaneous magnetic stimulation (a new, non-invasive method of modulating a patient’s nervous system activity) that targeted the left stellate ganglion was no better than a sham procedure in preventing VT recurrence within 24 h but was more effective in reducing VT burden (incidence rate ratio, 0.42; *P* < 0.001) and number of AADs.^212^

##### 8.2.8.2. Thoracic epidural anaesthesia

Thoracic epidural anaesthesia provides greater sympatholysis, as administration of an anaesthetic in the C7-T4 epidural space can block sympathetic activity along the stellate and thoracic sympathetic ganglia bilaterally. This procedure is performed under fluoroscopic guidance. Contraindications include active infection, dual anti-platelet therapy, and the requirement for uninterrupted anticoagulation. Similar to SGB, TEA has been shown to reduce VT burden in small case series of patients with VT storm or refractory VT by ≥80%, though prospective and randomized data are lacking.^213,214^ Notably, SGB and TEA are temporary measures used as a bridge to more definitive therapy, such as CA, CSD, or occasionally, heart transplantation.

##### 8.2.8.3. Surgical/thoracoscopic sympathetic cardiac denervation

The role of surgical CSD has been studied in patients with LQTS and CPVT (see Section 6.4.), as well as in those with refractory VT/VF storm and SHD.^215^ Left CSD (LCSD) effectively reduces the burden of VT in LQTS and CPVT patients and has been used in cases of BB intolerance or refractoriness.^216–220^ In a series of 147 high-risk LQTS patients, 75% of whom were symptomatic despite BB therapy, and the mean yearly number of cardiac events, including syncope, was reduced by 91% after CSD.^219^ Bilateral CSD has shown better results in patients with SHD,^221–225^ and in the largest multi-centre series of 121 patients (mean LVEF 30 ± 13%, 71% with NICM, 75% with VT storm, and 92% on BB combined with an AAD), bilateral CSD was associated with a 58% 1-year ICD shock/transplant-free survival and a >90% reduction in ICD shock burden in the year after compared with the year prior to CSD.^224^ In another series of 21 patients of which >70% had presented with ES, 76.9% were free from recurrent ICD shocks and 38.5% were free from any VA during a mean follow-up of ∼9 months.^215^ Notably, in patients with SHD, CSD may be efficacious in both monomorphic VT and PVT or VF,^215^ and utilization of CSD at an early stage, compared with performing the procedure urgently or emergently, may lead to better outcomes.^226^ Cardiac sympathetic denervation is currently being evaluated in randomized clinical trials to treat refractory VT (NCT01013714). A retrospective study of patients undergoing CSD suggested that CSD may also reduce the burden of PVCs, potentially playing a role in reducing triggers for VT/ES.^227^

##### 8.2.8.4. Renal denervation

Renal denervation involves the ablation of renal nerves surrounding the renal arteries. This procedure can be performed under electroanatomic mapping and fluoroscopic guidance.^228,229^ In several case series of patients with cardiomyopathy, including DCM and Chagas cardiomyopathy, RDN with or without VT ablation was reported to reduce the burden of VT.^230–232^ In a meta-analysis of 121 pooled patients from multiple studies/case series (mean LVEF of 30.5 ± 10.3%, 76% men, 99% on BB, and 79% on amiodarone therapy), RDN reduced ICD shocks and ATP episodes.^229^ Primary challenges with RDN include a lack of clear procedural endpoints and anatomic variability of structures surrounding the arteries that can affect ablation biophysics and efficacy.^233^ Prospective randomized data are lacking.

**Advice Table 6:** Sedation and autonomic modulation

	Evidence	Strength
**Advice TO DO**		
Initiate sympathetic blockade (esmolol, propranolol) in preference to lidocaine i.v. in ES patients with recent (<3 months) MI^26^	RCT	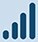
The combination of i.v. amiodarone and oral propranolol is preferred to the combination of i.v. amiodarone and oral metoprolol in the management of ES in ICD patients^149^	RCT	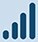
Initiate mild-to-moderate sedation (benzodiazepine) in all patients with an ES and ongoing/recurrent arrhythmia^140,141^	OBS	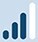
**May be appropriate TO DO**		
Deep sedation/general anaesthesia and mechanical ventilation may be appropriate in case of drug-refractory ES^140,141^	OBS	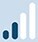
Stellate ganglion block or TEA may be appropriate in SHD in the setting of ES or incessant VT/VF to reduce burden of VAs and ICD shocks as a bridge to more definitive therapy^26,205–207,213^	OBS	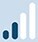
**Areas of uncertainty**		
The role of RDN as adjunct to ablation in patients with recurrent VAs and ES is uncertain	OBS	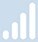

#### 8.2.9. Overdrive pacing

Overdrive pacing (ODP) may extinguish abnormal ventricular automaticity by overriding the ectopic pacemaker, which permits capturing and suppressing the ectopic pacemaker. Overdrive pacing may also cause an exit block from the ectopic focus. In re-entry VT, fast pacing may disrupt the re-entry circuit by altering the conduction rate, changing the excitation pathway, shortening, and decreasing dispersion of refractoriness of tissues in the re-entrant circuit and restitution of more uniform repolarization.^234^ By reducing ventricular refractory period, ODP may also reduce the susceptibility to the R on T phenomenon. These effects can be augmented by addition of AAD.^235^

Overdrive pacing can be carried out with an implanted pacemaker or ICD or through a temporary electrode inserted into the RV, by pacing at a rate of 10–20 b.p.m. greater than the native rate. Of note, decremental ODP (with progressively shorter inter-stimuli intervals) was superior to fixed overdrive burst pacing (with constant intervals) in interrupting VT and auto-decremental pacing (a 10 ms decrement) and was superior to scheme using a 5 ms coupling decrement.^236,237^ Temporary overdrive atrial or ventricular pacing in patients without ICD demonstrated its efficiency in preventing recurrence of drug-resistant VA in several case reports, mainly in the context of acute or recent myocardial ischaemia.^238,239^ Moreover, bradycardia pacing might be useful in some patients with brady-mediated PVT.

#### 8.2.10. Bailout strategies

The inability to create transmural or deep intramural lesions in the ventricular myocardium is one of the reasons for VT CA failure. One solution is sequential radiofrequency applications from adjacent opposite sites, including epicardial access. However, this might not be sufficient, and several bailout strategies have been proposed and described in the literature.^240–248^ These optional strategies include prolonged or high-power applications, bipolar ablation, simultaneous unipolar ablation, use of low-ionic solution (half normal saline), retractable needle ablation, septal coronary venous mapping, intracoronary artery wire mapping/ablation, ethanol ablation, coil embolization, or stereotactic radiotherapy. All these techniques have not been utilized in large or randomized series and have been described/adopted by highly skilled operators working in high-volume centres.

## 9. Stabilized patient

### 9.1. Long-term treatment

Without specific anti-arrhythmic interventions, ES recurs in >50% of patients with SHD. The risk of ES recurrence is highest within the first year after the event. Independent predictors of ES recurrence were LVEF ≤30%, age >65 years, and lack of optimal HF treatment in one observational study.^249^.

Beta-blockers,^120^ angiotensin-converting enzyme (ACE) inhibitors or angiotensin receptor blockers (ARBs),^250^ spironolactone,^251^ and angiotensin receptor-neprilysin inhibitor (ARNI; valsartan–sacubitril)^252^ have been associated with a reduced risk of arrhythmia and SCD in patients with HF with reduced LVEF (HFrEF). There is limited evidence that statins^253^ may reduce the occurrence of VA. Preliminary data suggest a beneficial effect of sodium-glucose co-transporter-2 (SGLT-2) inhibitors.^254^ Although there are no data evaluating the effect on ES recurrence specifically, optimization of HF treatment is important and should follow current guidelines.^255^ In patients with cCAD, complete revascularization may be beneficial.^256^

Precipitating factors, including potential pro-arrhythmic drugs, excessive alcohol consumption,^257–259^ illicit drugs,^260^ and electrolyte imbalances, must be avoided.

### 9.2. Anti-arrhythmic drugs to prevent electrical storm recurrence

#### 9.2.1. Chronic anti-arrhythmic drug therapy in patients with structural heart disease

Randomized controlled trials or large observational studies evaluating the efficacy of AAD to prevent specifically ES recurrence are scarce. However, the potential efficacy may be extrapolated from studies that addressed the efficacy of AAD in preventing VT recurrence in general. Anti-arrhythmic drugs used to prevent ES recurrence are listed in *Table 11*.

**Table 11:** Pharmacotherapy for chronic treatment of patients with ES

Chronic treatment									
Vaughan Williams class	Drug	Channels effected	Dose (p.o.)	Half-life	Metabolism	Specific use	Adverse effect	Monitoring	Counter indications/warnings
Class I	Quinidine	I_Na_, I_Kr_, I_to_, M, α	600–1600 mg Loading dose: start 200 mg every 3 h until effect, maximum 3 g in first 24 h	6–8 h	Hepatic	VT, VF, IVF, BrS, SQT	QTc prolongation, TdP, QRS prolongation, increases DFT, hypotension, bradycardia HF exacerbation, diarrhoea, immune thrombocytopenia	ECG (QRS duration and QT interval), heart rate, platelet count	Severe bradycardia and/or high-degree AV block in the absence of a pacemaker, myasthenia gravis Use with caution in HF
	Procainamide	I_Na_, I_Kr_ ganglionic block	500–1250 mg q6 h	3–4 h	Hepatic and renal	VT, pre-excited AF	QTc prolongation, TdP, QRS prolongation, increases DFT, lupus-like syndrome, bone marrow aplasia	ECG (QRS duration and QT interval), blood pressure	Myasthenia gravis Use with caution in HF
	Disopyramide	I_Na_, I_Kr_,M	250–750 mg	4–10 h	Hepatic	VT, PVCs	Negative inotrope, QRS prolongation, AV block, pro-arrhythmia (atrial flutter, monomorphic VT, TdP), anticholinergic effects	Heart rate, blood pressure, ECG (QRS duration, QT interval)	Severe bradycardia and/or high-degree AV block and/or severe intraventricular conduction delay in the absence of a pacemaker, decompensated HF, congenital QT prolongation, myasthenia gravis
	Mexiletine	I_Na_	600–1200 mg Loading dose: 400 mg initially followed by 600 mg in the first 24 h	10–14 h	Hepatic	VT/VF, LQTS3 LQT2	Tremor, nausea, paresthesia, numbness	ECG (QT interval), hepatic function	
	Flecainide	I_Na_	200–400 mg	12–27 h	Hepatic	VT, PVCs, CPVT	QRS prolongation, negative inotrope, AV block, sinus bradycardia, pro-arrhythmia (atrial flutter, monomorphic VT, occasional TdP), increase pacing threshold	Heart rate, blood pressure, ECG (QRS duration, QT interval)	Severe bradycardia and/or high-degree AV block and/or severe intraventricular conduction delay in the absence of a pacemaker, ischaemia, SHD, BrS, myasthenia gravis
Class II	Metoprolol	β1-receptor	25–400mg	Tartarate: 3–4 h Succinate: 3–7 h	Hepatic	VT, PVCs	Bradycardia, AV block, hypotension, nausea, dizziness	Heart rate, blood pressure	Severe bradycardia and/or high-degree AV block in the absence of a pacemaker, decompensated HF, cardiogenic shock, myasthenia gravis
	Propranolol	Non-selective BB	80–320 mg	3–6 h	Hepatic	VT, PVCs, LQTS	Bradycardia, AV block, hypotension, nausea, dizziness bronchospasm		Severe bradycardia and/or high-degree AV block in the absence of a pacemaker, decompensated HF, cardiogenic shock, myasthenia gravis
	Nadolol	Non-selective BB	40–120 mg	20–24 h	Renal	VT, PVC, LQTS, CPVT	Bradycardia, AV block, hypotension, nausea, dizziness, bronchospasm		Severe bradycardia and/or high-degree AV block in the absence of a pacemaker, decompensated HF, cardiogenic shock, myasthenia gravis
Class III	Amiodarone	I_Na_, I_Ca_, I_Kr_, I_K1_, I_Ks_, I_to_, α, β	200–400 mg Loading dose: 600–1200 mg/ 24 h 8–10 days	4–14 weeks	Hepatic	VT, VF, PVCs	Increases DFT, hypotension, bradycardia, AV block, QT prolongation, TdP (rare), hypothyroidism/hyperthyroidism, nausea, photosensitivity, skin discolouration, peripheral neuropathy, tremor, hepatitis, pulmonary fibrosis/pneumonitis	Heart rate, blood pressure, ECG (QT interval), hepatic function	Severe bradycardia and/or high-degree AV block in the absence of a pacemaker, decompensated HF, cardiogenic shock
	Sotalol	I_Kr_, β	160–640 mg	12 h	Renal	VT, VF, PVCs	Bradycardia, AV block, QT prolongation, TdP, hypotension, nausea, dizziness, bronchospasm	Heart rate, blood pressure, ECG (QT interval)	Severe bradycardia and/or high-degree AV block in the absence of a pacemaker, decompensated HF, cardiogenic shock
Class IV	Verapamil	I_Ca_	120–480 mg	3–7 h		Fascicular VT	Bradycardia, AV block, negative inotrope, hypotension	Heart rate, blood pressure, hepatic function	Severe bradycardia and/or high-degree AV block in the absence of a pacemaker, decompensated HF, cardiogenic shock, myasthenia gravis

BB, beta-blocker; BrS, Brugada syndrome; CPVT, catecholaminergic polymorphic ventricular tachycardia; ECG, electrocardiogram; ES, electrical storm; HF, heart failure; IVF, idiopathic ventricular fibrillation; LQT, long QT; LQTS, LQT syndrome; PVC, premature ventricular complex; QTc, corrected QT; SHD, structural heart disease; SQT, short QT; TdP, torsade de pointes; VF, ventricular fibrillation; VT, ventricular tachycardia.

Beta-blockers are a cornerstone of drug treatment of patients with VA in the context of SHD. In a large retrospective registry of VT/VF survivors, chronic BB use was associated with improved survival at 3 years compared with a propensity-matched group not taking BBs.^261^

Amiodarone, preferably in combination with BB, can reduce VA recurrence and ICD shocks. Several studies and meta-analyses have demonstrated its superior anti-arrhythmic effect in reducing recurrent VTs or appropriate ICD therapies compared with other AADs.^208–211^ In a recent meta-analysis of 22 RCTs, including 3828 patients, amiodarone use was associated with a 70% relative risk reduction for VT recurrence [hazard ratio (HR) = 0.34; 95% confidence interval (CI) = 0.15–0.74] and appropriate ICD therapies (HR = 0.33; 95% CI = 0.15–0.76), compared with standard optimal medical therapy, including BBs, in patients with ICD and a history of VA.^262^ In the Optic study, the combination of BB and amiodarone was superior to sotalol or BB monotherapy in reducing the risk of appropriate ICD shocks during 1 year observation in ICD patients implanted for secondary prevention.^263^ However, treatment-attributed adverse events need to be considered, including hypothyroidism and hyperthyroidism, pulmonary and liver toxicity, and drug-related mortality.^264^ In a recent RCT meta-analysis, amiodarone use was associated with an increased risk of death (odds ratio 3.36, 95% CI 1.36–8.30, *P* = 0.009).^265^ Similarly, in a large observational study of 281 patients with DCM undergoing RFCA for drug-refractory VT (ES 35%, incessant VT 20%), amiodarone at discharge was independently associated with mortality or heart transplantation (HR 3.23, 95% CI 1.43–7.33, *P* < 0.01).^266^ Whether this is directly amiodarone related or whether amiodarone is just a marker for sicker patients is not clear yet.

Sotalol is a racemic mixture of two isomers, of which the d-isomer blocks potassium channels and the l-isomer acts as a non-selective BB together with Class 3 properties. Lower doses exert mainly a beta-blocking effect, whereas higher doses are needed for the Class III effect; however, significant QT prolongation has been observed after a single low dose.^267,268^ In the Optic trial, sotalol was superior to BBs alone in reducing the risk of ICD shocks.^263^ Of note, the number of all episodes terminated by ATP or ICD shock was not significantly different between patients on sotalol or BBs.^262^ In the d,l-Sotalol Implantable Cardioverter-Defibrillator randomized trial, sotalol reduced the mean number of ICD shocks compared with placebo (1.43 vs. 3.89 per year, *P* = 0.008), irrespectively from the LVEF.^269^ However, pro-arrhythmic side effects, including TdP, are of concern, and careful monitoring of the QTc interval, in particular in patients with impaired renal function, is mandatory. Sotalol should be used with caution in patients with HF and poor LVEF due to its negative inotropic effect. However, it may be useful in patients with ARVC.

Mexiletine is a Class IB AAD with high bioavailability (80%) that allows for its oral administration. It acts by blocking rapid sodium channels and was used as adjunctive therapy to amiodarone to prevent VA in case of amiodarone inefficacy or intolerable side effects.^270–272^ There are little data on the long-term safety of its use in patients with advanced SHD. Rapid loading (1 × 400 mg per os) was associated with HF decompensation in patients with severe LV dysfunction.^273^ It has also been shown to increase DF thresholds.^274^

Procainamide is a Class IA AAD, prolonging APD at faster rates (through the blockade of the rapid inward Na depolarization current in a use-dependent fashion) and prolonging repolarization via the blockade of the delayed rectifier K channel. It reduced spontaneous VA and suppressed VT inducibility in the ESVEM Trial and small, single-centre studies.^275,276^ In a recent small single-centre observational study, oral procainamide reduced ICD therapies in patients with VT refractory to other AADs, including amiodarone.^277^ However, severe side effects, including a drug-induced lupus erythematosus-like syndrome, are of concern. Of note, procainamide is not available in many countries.

Quinidine is a Class IA AAD, available in oral formulations, which has been shown to have only a modest negative inotropic effect.^278^ Quinidine plays a role in suppressing PVC-induced PVTs and VF in the subacute phase of MI (but also after revascularization: CABG and PCI) and in patients without SHD.^150,197^ However, quinidine showed limited tolerability and long-term efficacy when used in the management of amiodarone-refractory scar-related MSVT.^279,280^

While flecainide should be avoided in patients with cCAD or HFrEF,^281,282^ it can be effective, in combination with BBs or sotalol, in preventing recurrent VT in patients with ARVC refractory to single AAD or ablation.^283,284^ However, there is also the potential risk of myocardial depression and right (and left) ventricular failure in these patients, so caution is mandatory also in this population.

#### 9.2.2. Chronic pharmacotherapy in primary electrical disease

Beta-blockers remain the cornerstone of chronic treatment in LQT syndromes, and non-selective agents (propranolol, preferably long-acting and nadolol, usually starting from doses 80 and 40 mg 1× daily, respectively) proved to be more effective.^285^ Of note, the effectiveness of BBs not only as a group but also of a given drug (e.g. nadolol and propranolol > atenolol and metoprolol) might depend on the LQTS genotype (LQT1 > LQT2 ≥ LQT3).^286^

Mexiletine or ranolazine can be useful in Type 3 LQTS and mexiletine probably also in other LQTS subtypes.^287–289^ If adequate ICD interventions or ES episodes recur despite optimal beta-blockade, LCSD was shown to protect against VA in LQTS effectively (see Section 8.2.8.).^216,218,219^

The role of pharmacotherapy is limited in BrS, but quinidine was beneficial in preventing VA (start dose is 250 or 300 mg bid), ES, and frequent ICD shocks.^290^ In the randomized QUIDAM study, hydroquinidine seemed to be effective in preventing life-threatening VA, however, at the cost of frequent (68%, mainly gastrointestinal) side effects.^291^

Catecholaminergic polymorphic ventricular tachycardia respond well to BBs; those who experience recurrent syncope or bidirectional VT while on BBs may benefit from adding flecainide.^292–294^ Left cardiac sympathetic denervation is an option if VA recur despite drugs.^216^ In ERS and IVF patients with ES, quinidine may prevent VA.^197^ Long-term treatment with hydroquinidine was efficient in short QT syndrome, but data are limited.^295,296^

Implantable cardioverter-defibrillator treatment is indicated, in general, in secondary prevention or case of arrhythmic syncope unless there is a clear unequivocal trigger underlying the arrhythmias (i.e. drug use in LQTS).^1^

### 9.3. Catheter ablation to prevent electrical storm recurrence

Catheter ablation for VA is increasingly performed and offers an alternative to AAD treatment, in particular, if AAD are not tolerated, not effective, or not desired. Most of the data on ablation outcome are derived from patients with cCAD, DCM subgroups, or ARVC who were treated in highly experienced centres.

Procedural risks and expected outcome depend on individual patient characteristics (including the specific underlying aetiology, substrate location and extent, comorbidities, prior surgery, and prior ablation attempts), and the experience of the treating centres and operators. It is adviced to provide the patient with detailed information on the procedural aspects, risks, and expected outcomes for shared decision-making.

#### 9.3.1. Ischaemic heart disease

While ischaemic heart disease (IHD) represents one of the most common ES substrates, limited data are available addressing specifically the benefit of CA after ES stabilization. A systemic review of randomized clinical trials, including the SMS, VANISH, CALYPSO, VTACH, SMASH VT trials, and the more recent BERLIN VT trial, showed that CA significantly reduces the occurrence of ES and appropriate ICD therapies, including ICD shocks.^297–301^ Of note, CA was efficient in controlling VA resistant to AADs^302,303^ and was more effective than amiodarone escalation or adjunctive mexiletine.^264,299,300,304,305^ A recent observational study demonstrated that early CA appears superior to initial medical therapy regarding VT and ES recurrence, iatrogenic complications, cardiovascular hospitalizations, and cumulative days in the hospital.^306^ There are insufficient data to evaluate mortality reduction by CA in patients with ES. While CA did not significantly improve survival or cardiovascular hospitalizations in a meta-analysis of RCTs, non-randomized studies have reported survival benefits.^14,186,307,308^ In stabilized patients with cCAD, who present with an ES on chronic amiodarone therapy RFCA is advised.^299,300^ In patients without chronic amiodarone therapy CA may be appropriate dependent on patient’s characteristics, comorbidities, and preferences.^187^

#### 9.3.2. Non-ischaemic cardiomyopathy

Patients presenting with ES from NICM comprise a heterogeneous group of different phenotypes, including DCM, HCM, and ARVC phenotypes. In particular, DCM can be due to an inherited or acquired aetiology. Whether CA is beneficial to prevent ES recurrence in patients with NICM is unknown. Patients with NICM undergoing VT ablations had higher VA recurrence compared with patients with cCAD.^309^ Among the different non-ischaemic aetiologies, outcome after RFCA differs and was poorer in patients with HCM, valvular heart disease, (active) CS, and inherited DCM. This is often attributed to a more complex VA substrate (e.g. more intramural, epicardial, and septal involvement) as well as disease progression.^310–312^. Observational data suggest that CA may be associated with lower in-hospital mortality compared with medical management in selected patients.^313^

Whether to pursue endocardial or epicardial ablation can vary according to the specific aetiologies and suspected disease substrates. Patients with ARVC might benefit more from the epicardial–endocardial ablation strategy.^314^ Combined epicardial–endocardial ablation may also be considered at the index procedure in VTs caused by Chagas disease, as the epicardial scars are often more extensive than endocardial scars with a high number of epicardial circuits.^315,316^ Ventricular tachycardia ablation in stabilized NICM patients presenting with an ES under chronic AAD treatment may be appropriate. In individual patients with ARVC, chronic myocarditis, or VT substrate location that are expected to be approachable by RFCA based on pre-procedural evaluation, it is appropriate to carefully discuss this treatment option as an alternative to chronic AAD treatment.

However, considering the complex procedure, with often required epicardial–endocardial approach and bailout strategies, in particular for deep intramural substrates, transferring these patients to high-volume, experienced centres is advised.

#### 9.3.3. Primary electrical disease

In PED, CA is not a first-line option. Recent advancements, however, raised interest in the ablation of an epicardial arrhythmic RVOT substrate in BrS. The sentinel study by Nademanee *et al.,*^317,318^ demonstrating epicardial RVOT CA as a viable option in high-risk symptomatic patients, was followed by a series from Italy and a long-term follow-up study of an international cohort.^319,320^ These studies showed a high success rate (also in the long term) and low complication rates. It should, however, be noted that these procedures were performed in highly experienced, high-volume centres. Before considering prophylactic CA of symptomatic or even asymptomatic patients as an addition to ICD placement, further research including prospective randomized study is required.^321^

Preliminary reports of successful CA in patients with LQTS have been published.^322^ In three of the four patients who underwent mapping, the ectopy originated from the specialized conduction system in the LV (ramifications of the anterior and posterior fascicles). In a recent study, Pappone *et al.*^323^ identified electrophysiological abnormalities in the RV epicardium in a small series of LQTS patients. Eliminating these abnormal electrical activities successfully prevented malignant VA recurrences during a mean follow-up period of 1 year. Adoption of this approach requires further research.

In CPVT, recent experimental studies suggest a Purkinje fibre-related origin of the ventricular ectopy.^324^ However, clinical studies in occasional patients are inconclusive, demonstrating Purkinje fibre origin in some, but not all cases.^63^

In drug-resistant cases on ERS and IVF, in whom similar PVCs trigger VF, CA may be pertinent if performed in experienced centres.^325^

### 9.4. Autonomic modulation

Electrical storm recurrences are common, especially within the first year after the acute event. The pro-arrhythmic effect of sympathetic–parasympathetic imbalance may still play an important role beyond the acute phase,^249,326^ and initiation of long-term BB therapy is appropriate.^26,149^

While SGB and TEA are usually considered as acute interventions^327^ with a transient effect, surgical or thoracoscopic left or bilateral CSD results in permanent afferent and efferent CSD (see Section 8.2.8.).

**Advice Table 7:** Chronic treatment of patients with SHD who present with an ES after initial stabilization

	Evidence	Strength
**Advice TO DO**		
Optimize HF treatment in patients with HFrEF, including BBs, ACE inhibitors/ARB/ARNI, MRAs, and SGLT-2 inhibitors, according to the latest ESC Guidelines on HF management^120,250–252,254^	RCT	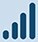
Perform CA, rather than AAD drug escalation in cCAD patients, if ES due to MSVT has recurred on chronic amiodarone therapy^264,299^	RCT	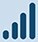
Perform CA in patients with cCAD and ES due to MSVT if AADs are contraindicated, not tolerated, or not desired^302,303^	OBS	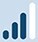
**May be appropriate TO DO**		
Long-term treatment with amiodarone and BB (choosing BB depending on clinical context) may be useful to prevent ES recurrence^263,265,328^	RCT	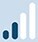
It may be appropriate to offer CA as first-line treatment in cCAD patients with ES due to MSVT, provided that the procedure is performed in experienced centres^185,301,306^	OBS	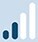
It may be appropriate to perform CA in patients with NICM, if ES due to MSVT recurs despite AAD therapy and if AADs are contraindicated or not tolerated, provided the procedure is performed in experienced centres^185,313^	OBS	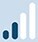
It may be appropriate to perform left or bilateral surgical or thoracoscopic CSD, to reduce burden of MSVT and PVT in ischaemic and NICM in patients with arrhythmia recurrences/ES despite medical therapy and CA and/or those who are not candidates for CA^213,224–226^	OBS	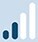

**Advice Table 8:** Chronic treatment of ES in stabilized primary electrical disease

	Evidence	Strength
**Advice TO DO**		
Perform LCSD in LQTS or CPVT patients with an ICD who are symptomatic on BB and who experience multiple shocks or ES refractory or intolerant to medical therapy^216–219^	OBS	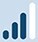
**May be appropriate TO DO**		
Quinidine therapy may be appropriate in patients with BrS who have recurrent ICD shocks or ES^290,291,329^	OBS	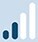
Quinidine in addition to an ICD may be appropriate for ES or recurrent VF in ERS, PVC triggered VF, or IVF patients^197,325^	OBS	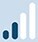
Catheter ablation by experienced electrophysiologists may be appropriate in patients with ERS or IVF with recurrent episodes of VF triggered by a similar PVC non-responsive to medical treatment^197,325^	OBS	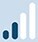
Catheter ablation of triggering PVCs and/or RVOT epicardial substrate may be appropriate in BrS patients with ES or recurrent appropriate ICD shocks^317–319^	OBS	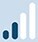

## 10. Psychological counselling

Symptoms of depression and anxiety are observed in 24–87% of ICD patients and impact the mental and functional quality of life.^330^ Both the concern of receiving an ICD shock and the experience of ICD shocks contribute to psychological distress. Young age (<50 years), frequent ICD shocks, and poor understanding of their cardiac condition or their device have been associated with increased distress.^330^ In a recent subanalysis from the Danish Trial, shocks during follow-up were associated with depression, anxiety, and ICD concerns.^331^ Depression and anxiety are associated with increased mortality in HF patients,^332^ and anxiety was associated with an increased risk of VAs in ICD patients.^333^ As a consequence, psychological counselling is advised as part of the multi-disciplinary care for ES patients. Noteworthy, in a recent EHRA-initiated survey, only 46% of the surveyed European electrophysiology (EP) centres refer patients to psychological counselling after ES.^334^

**Advice Table 9:** Informing the patient, end-of-life issues, and psychological councelling

	Evidence	Strength
**Advice TO DO**		
Provide the patient with detailed information on the procedural aspects, risks, and expected outcomes for shared decision-making, before undertaking CA or any invasive treatment, if possible	OPN	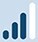
Psychological counselling is advised as a part of the multi-disciplinary care for ES patients^331,335^	OBS	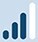
In the presence of significant comorbidities, particularly in the case of recurrent ES and intractable, end-stage HF, informed discussion with the patient and family about the options of ICD deactivation should be undertaken	OPN	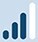

## 11. Special patient groups

### 11.1. Patients with left ventricular assist device

Ventricular arrhythmias and ES are common in patients with implanted LV assist device (LVAD), with reported incidences of 20–50 and 9%, respectively.^336–338^ Ventricular arrhythmia in patients with LVADs are usually well tolerated, especially in those with continuous flow LVAD. However, short-term mortality after an ES has been reported to be high, and recurrent ICD shocks while conscious are of concern.^338–340^ Ventricular arrhythmia in LVAD patients can be occasionally due to suction, which may be managed by changing the turbine speed.^341^ Mapping studies have demonstrated that the majority of MSVT is not related to apical inflow cannula, but rather to remote intrinsic myocardial scar.^341,342^ Anti-arrhythmic drug treatment, sedation, and autonomic modulation to control the ES do not differ between patients without or with LVADs.

Small case series from very experienced groups have reported favourable outcomes after RFCA in highly symptomatic patients with ES or recurrent ICD shocks, usually after the failure of AAD.^341,342^ Practical considerations include the following: the lack of pulsatile peripheral flow during vascular access and blood pressure monitoring and the potential absence of aortic valve opening, favouring transseptal access. A theoretical risk of catheter entrapment in the LVAD cannula/impeller and considerable noise interference on surface ECG and mapping from HeartMate 3 should also be taken into account.^343^ The mechanical haemodynamic support provided by the LVAD usually allows the advised transfer of patients to specialized centres experienced in the management of patients with LVADs and VA.

### 11.2. Patients with advanced heart failure

Patients with advanced HF are at high risk for VA,^344^ HF-related recurrent hospitalizations, and mortality. Ventricular arrhythmia and ES can be the consequence of end-stage HF but can also worsen cardiac function. Optimization of HF treatment in close collaboration with the HF specialist and cardiothoracic surgeon is mandatory to identify those who benefit from advanced HF treatment, including LVAD and heart transplantation. Anti-arrhythmic drug treatment is limited because of the negative inotropic effect of almost all AAD. Amiodarone and mexiletine, in selected cases, can be effective. Catheter ablation offers an alternative, particularly if substrate-based approaches are used, and recurrent VT induction is avoided. Patients with severe HF symptoms at rest (NYHA IV) are usually excluded from RCTs evaluating CA or are combined with patients with NYHA Class III. While VT ablation did not reduce mortality in patients with cCAD, it was superior to the escalation of anti-arrhythmic medication in reducing VT recurrences and ES during follow-up.^264^ The benefit was consistent across functional Class I through III. In a large international VT ablation registry of 1365 patients, 111 patients in NYHA IV were included.^345^ Although there was no significant difference in acute complications, NYHA IV patients required more haemodynamic support and had a higher in-hospital mortality. Patients with advanced HF have a higher risk of acute decompensation during VT ablation, which is associated with high mortality risk.^178^ Patients with advanced HF require a multi-disciplinary team to decide on the acute management of the ES and whether they are candidates for MCS (see Section 8.2.6.), LVAD (see Section 11.1.) or heart transplantation. Early evaluation for advanced HF management is advised for patients with DCM, LVEF ≤32%, and early VT recurrence after initial ablation for an ES, considering the high mortality independent from the arrhythmic outcome.^266^

## 12. When not to perform interventional treatment

In terminally ill patients or advanced cardiac disease, the type of treatment, the expected procedural success of invasive treatments, and the willingness and goals of care for the patient and family are important to consider. In the presence of significant comorbidities, particularly in the case of recurrent ES, a holistic approach and informed discussion with the patient and family about the options of ICD deactivation and treatments to relieve distress and provide patient comfort are advised.
